# Silencing of Long Non-coding RNA NEAT1 Upregulates miR-195a to Attenuate Intervertebral Disk Degeneration via the BAX/BAK Pathway

**DOI:** 10.3389/fmolb.2020.00147

**Published:** 2020-08-11

**Authors:** Ning Tang, Yulei Dong, Jiaming Liu, Hong Zhao

**Affiliations:** ^1^Department of Orthopedics, Peking Union Medical College Hospital, Chinese Academy of Medical Sciences, Beijing, China; ^2^Department of Orthopedics, The First Affiliated Hospital of Nanchang University, Nanchang, China

**Keywords:** NEAT1, miR-195a, BAX/BAK, extracellular matrix, intervertebral disk degeneration

## Abstract

**Background/Aims:**

An increasing body of evidence has demonstrated that long non-coding RNAs (lncRNAs) play a vital regulatory role in intervertebral disk degeneration (IVDD). Nucleus enriched abundant transcript 1 (*NEAT1*), a novel cancer-related lncRNA, is associated with many malignancies, including ovarian cancer, and esophageal squamous cell carcinoma. Nevertheless, the role of *NEAT1* in the progression of IVDD remains to be studied. Here, we explored the effect of *NEAT1* on the progression of IVDD and the mechanisms involved.

**Methods:**

An IVDD model was constructed in SD rats *in vivo*, and degeneration was induced by advanced glycation end product (AGE) in human nucleus pulposus cells (HNPC) *in vitro*. Quantitative real-time PCR was performed to detect the relative *NEAT1* and miR-195a expressions and further confirmed the relationship between *NEAT1* and miR-195a. Cell apoptosis was evaluated by TUNEL assay. The related mechanisms were explored by Western blot assay.

**Results:**

The relative *NEAT1* expression was significantly upregulated in the IVDD rat model and the denatured HNPC. Silencing of *NEAT1* expression in HNPC significantly promoted the Collagen II and TIMP-1 expression induced by AGE while greatly suppressing the expressions of MMP-3 and cleaved caspase-3. Besides, downregulation of *NEAT1* obviously reversed the AGE-induced apoptosis in HNPC. More interestingly, these effects of *NEAT1* knockout on HNPC were largely reversed by silencing of miR-195a or overexpression of BAX under the AGE treatment. Mechanically, the direct combination of *NEAT1* with miR-195a resulted in upregulation of MMP-3, cleaved caspase-3, BAX, and BAK, as well as downregulation of Collagen II and TIMP-1, which are associated with EMT and apoptosis. We also demonstrated similar results in the *in vivo* experiments.

**Conclusion:**

*NEAT1* played its role in IVDD progression via partly by mediating the miR-195 expression and might be used as a potential target for IVDD therapy.

## Introduction

As the pathological basis of spinal degenerative diseases, intervertebral disk degeneration (IVDD) is often caused by many factors, including genetic factors, immune factors, matrix metalloproteinases (MMPs), inflammatory mediators and extracellular matrix (ECM) factors, aging, mechanical load, etc. (Vergroesen et al., 2015; Dowdell et al., 2017). However, the exact mechanism of IVDD has still not been illuminated. Recently, researchers have tried to delay or reverse the progression of IVDD to reduce the occurrence of disk degeneration (Chen S. et al., 2019). Hence, exploring targeted genes in blocking the pathological progression of IVDD is of great significance.

Long-chain non-coding RNA (LncRNA) has been proved to be involved in regulating various biological processes. Meanwhile, IVDD is a complex process involving genetic factors, biomechanics, apoptosis, and other internal and external factors that ultimately lead to pathological changes (Dowdell et al., 2017; Mohanty and Dahia, 2019). Differentially expressed long non-coding RNAs (lncRNAs) in intervertebral disk tissues play a vital role in the progression of IVDD by regulating the expression of target genes to affect the proliferation and apoptosis of nucleus pulposus (NP) cells, changes in the ECM, and other physiological processes (Chen W.K. et al., 2017; Wang et al., 2017; Li et al., 2018). Chen J. et al. (2017) have proved that lncRNA *TUG1* can suppress the apoptosis and senescence of human NP cells and improve the imbalance of ECM metabolism induced by tumor necrosis factor-α (TNF-α) through regulating the Wnt-β-catenin signaling pathway. Besides, lncRNA *FAF1* can extend the S phase of NP cells through the ERK signaling pathway to promote cell proliferation (Mi et al., 2018). Nucleus enriched abundant transcript 1 (*NEAT1*), which is mainly located in the paraspeckle of nucleus, participates in regulating many genes. A previous lncRNA–mRNA microarray study showed that anomalous NEAT1 expression was higher in intervertebral disk degeneration (IDD) than in normal disks (Lan et al., 2016). Ruan et al. (2018) have demonstrated that *NEAT1* can participate in the degradation of EMT in NR cells through the MAPK-ERK1/2 signaling pathway, thereby accelerating the degeneration of disk tissues. However, how NEAT1 regulates the IVDD progression and the related molecular mechanism remain largely unknown.

Currently, more and more studies are focusing on the molecular mechanism by which lncRNA acts as a natural miRNA sponge or competes with other RNA transcriptors for specific miRNA, indirectly regulating the biological processes involving miRNA at the post-transcriptional level (Yang et al., 2019). In the regulation of IVDD, lncRNA acts as a miRNA sponge, which leads to a reduction in miRNAs involved in regulating downstream genes, thereby indirectly regulating the target gene expression and ultimately regulating the proliferation and apoptosis of NP cells and the formation of ECM (Shao et al., 2019). Wang et al. (2017) found that *RP11-296A18.3* promotes the expression of its neighboring coding gene *HIF1A* through competitive combination with miR-138 to accelerate the proliferation of NP cells and the synthesis of ECM. Xi et al. (2017) have proved that *HCG18*, upregulated in NP cells, competitively binds to miR-146a-5p to promote the expression of TARF6, the target gene of miR-146a-5p, thereby preventing NP cells from entering the S phase and playing a key role in inducing apoptosis, macrophage recruitment, and osteogenic differentiation. Tan et al. (2018) also have demonstrated that *SNHG1* can block the expression of cyclin 1 gene through competitive combination with miR-326 to affect the proliferation of NP cells. Therefore, we proposed to determine whether NEAT1 also plays the role of a miRNA sponge to regulate the functions of miRNAs.

Here, we found that NEAT1 was upregulated in IVDD *in vivo* and *in vitro*. Additionally, downregulation of NEAT1 significantly suppressed the apoptosis induced by advanced glycation end product (AGE) in NP cells, while silencing of miR-195a or overexpression of BAX obviously reversed this effect. Mechanically, knockdown of NEAT1 resulted in the upregulation of matrix metalloproteinase 3 (MMP-3), cleaved caspase-3, BAX, and BAK and the downregulation of Collagen II and tissue inhibitor of metalloproteinase 1 (TIMP-1), which are associated with EMT and apoptosis via partly mediating miR-195a expression.

## Materials and Methods

### Rat IVD Degeneration Model

Sprague-Dawley (SD) rats (3-month-old, female) were purchased from Beijing Weitong Lihua Laboratory Animal Technology Co., Ltd. (SYXK 2016-0006, Beijing, China). Rats were fed normally and adapted to the environment for 1 week before the experiment. The annulus fibrosus (AF) needle puncture method was used to construct the rat model of IVD degeneration (IVDD). Briefly, rats were fasted 1 day before operation and underwent general anesthesia (4% of chloral hydrate 10 mL/kg) by intraperitoneal injection. Then, the abdominal cavity was opened, and the C5-C6 intervertebral disk and endplate were exposed. A puncture was made parallel to the endplates, and a 21-gauge needle was inserted into the C5-C6 intervertebral disk segment parallel to the level of the AF, crossing the nucleus pulposus (NP) up to the contralateral AF. The needle penetration depth was full-thickness needling of the AF, and then the needle was rotated 360° twice and kept in place for 30 s. Here, rats (*n* = 60) were randomly divided into six groups (10 rats per group) as follows: control, punctured (IVDD), Sham, IVDD + si-NEAT1, IVDD + si-NEAT1 + miR-195a-inhibitors, and IVDD + si-NEAT1 + pcDNA3.1-BAX. NEAT1siRNA, NEAT1siRNA + miR-195a-inhibitors, and NEAT1siRNA + pcDNA3.1-BAX were injected into the C5-C6 disk of rats immediately after the establishment of the puncture model, respectively. In the sham group, the C5-C6 intervertebral disk was exposed without being punctured. Twenty-eight days after the operation, the rats were sacrificed, and the intervertebral disks were collected for further analysis.

### Cell Culture and Treatment

Human Nucleus Pulposus Cells (HNPC, Cat. No. 4800) isolated from the nucleus pulposus of human intervertebral disk were purchased from ScienCell Research Laboratories (Carlsbad, CA, United States). The Nucleus Pulposus cell medium (Cat. No. 4801), consisting of 500 ml basal medium, 10 ml fetal bovine serum (FBS, Cat. No. 0010), 5 ml Nucleus Pulposus Cell Growth Supplement (Cat. No. 4852), and 5 ml penicillin/streptomycin solution (Cat. No. 0503), were obtained from ScienCell Research Laboratories. HNPCs were cultured with Nucleus Pulposus cell medium in an atmosphere of 5% CO_2_ at 37°C. The culture medium was replaced every 2 days. According to the research needs, the cells were divided into eight groups: Control, AGE (100 μg/mL, 48 h); AGE + Negative Control (NC) siRNA; AGE + NEAT1 siRNAs; AGE + NEAT1 siRNAs + miR-195a-inhibitors; AGE + NEAT1 siRNAs + inhibitor-NC; AGE + NEAT1 siRNAs + pcDNA3.1-BAX plasmids; AGE + NEAT1 siRNAs + pcEV.

### Transfection

To change the expressions of NEAT1, miR-195, and BAX, HNPCs were transfected with NEAT1 siRNAs, 195a-inhibitors, or pcDNA3.1-BAX plasmids using Lipofectamine2000 (Invitrogen, United States) according to the instructions, respectively. The non-sense strand NC siRNA, NC-inhibitor, and pcEV were used as NC, respectively. Non-sense strand NC siRNAs, NEAT1 siRNAs, 195a-inhibitors, pcEV, and pcDNA3.1-BAX plasmids were obtained from RiboBio (Guangzhou, China). Real-time PCR and Western blot were used to detect the transfection efficiencies.

### Real-Time PCR

The expressions of NEAT1 and miR-195a were analyzed by real-time PCR. Trizol reagent (Takara, Dalian, China) was used to extract the total RNA from tissues or cells, and the reverse transcription kit (DBI, United States) was used to reverse-transcribe the RNA into cDNA according to the manufacturers’ instructions. Real-time PCR analysis was implemented on an Agilent Stratagene Mx3000P Sequence Detection System. The primer sequences are as follows: U6 forward primer: 5′ -CTCGCT TCGGCAGCACA-3′, U6 reverse primer: 5′ -AACGCTTCACG AATTTGCGT-3′; GAPDH forward primer: 5′-CCTCGTCTC ATAGACAAGATGGT-3′, GAPDH reverse primer: 5′-GG GTAGAGTCATACTGGAACATG-3′;miR-195a forward primer: 5′-GGGGTAGCAGCACAGAAAT-3′, miR-195a reverse primer: 5′-CAGTGCGTGTCGTGGAGT-3′; NEAT1 forward primer: 5′-TGGCTAGCTCAGGGCTTCAG-3′, NEAT1 reverse primer: 5′-TCTCCTTGCCAAGCTTCCTTC-3′. GAPDH and U6 were used as internal control for NEAT1 and miR-195a, respectively. The calculation method of the relative expression amount was 2^–ΔΔ*ct*^.

### Western Blotting

RIPA buffer (Beyotime, Shanghai, China) was used to extract total protein, and BCA Protein Assay kit (Thermo Fisher Scientific, Waltham, MA, United States) was used to determine the protein concentration. Total proteins (30 μg per lane) were subjected to 12% SDS-PAGE gels and then transferred onto PVDF membranes (Millipore, Billerica, MA, United States). The membranes were blocked with 5% non-fat milk for 1.5 h. Subsequently, membranes were incubated with primary antibodies against TIMP-1 (cell signaling Tech, 8946, 1:1000), MMP-3 (cell signaling Tech, 14351, 1:1000), Collagen II (Abcam, ab34712, 1:5000), cleaved-caspase-3 (Abcam, ab49822, 1:500), Bax (cell signaling Tech, 2772, 1:1000), Bak (cell signaling Tech, 12105, 1:1000), and GAPDH (Abcam, ab181602, 1:10000) at 4°C overnight. Next, the HRP-conjugated goat anti-mouse IgG and anti-rabbit IgG (Zhongshan Golden Bridge Biotechnology, Beijing, China) were added and incubated for 1.5 h at room temperature. Finally, the bands were developed using ECL-Plus reagent (Thermo Fisher Scientific, Waltham, MA, United States), and the results were examined using a Gel Imaging System. The relative expression levels of proteins were calculated by the Gel-Pro Analyzer (Media Cybernetics, United States).

### Hoechst Staining

Cells (5 × 10^4^ per well) that had undergone different treatments were seeded into six-well plates and were stained with Hoechst solution (Beyotime Company, Shanghai, China) for 5 min. The morphological changes of cell nuclei were observed via a fluorescence microscope (Olympus, Tokyo, Japan). The relative apoptotic rate of Hoechst-positive cells in each sample was calculated.

### Luciferase Reporter Assay

To further investigate the role of NEAT1 in regulating the degenerative HNPC cells through its targeted miRNAs, we used a bioinformatics tool (StarBase) to predict the target miRNA, and the binding sites between miR-195a and NEAT1 were found. To further confirm the interaction between miR-195a and NEAT1, the dual-luciferase reporter system was used to detect the interactions between miR-195a and NEAT1. Briefly, the wild-type (WT) 3′-untranslated region (3′-UTR) of NEAT1 containing the miR-195a binding sites (NEAT1-WT) or mutant NEAT1 3′-UTR (NEAT1-MUT) were amplified and cloned downstream of the firefly luciferase gene in psiCHECK-2 plasmids (Promega, Madison, WI, United States). miR-195a mimics or NC for miR-195a (RiboBio, Guangzhou, China) together with NEAT1-WT or NEAT1-MUT, were co-transfected into HEK 293T cells using Lipofectamine 2000 reagent, following the instructions. Luciferase activity 48 h post-transfection was detected by using the Dual-Luciferase Reporter Assay System (Promega, Madison, WI, United States).

### Nucleus/Cytosolic Fraction Isolation

Total RNA of cytoplasm and nucleus was extracted by cytoplasmic and nucleus RNA separation reagents (Thermo Fisher Scientific, Waltham, MA, United States) according to the product manual. Briefly, HNPC cells (1 × 10^7^) were firstly lyzed with hypotonic buffer (25 mM Tris–HCl, PH 7.4, 1 mM MgCl_2_, 5 mM KCl) and then an equal volume of hypotonic buffer containing 1% NP-40 was added. After centrifugation, the supernatant was collected as the cytosolic fraction. The pellets were re-suspended in nucleus resuspension buffer (20 mM HEPES, pH 7.9, 400 mM NaCl, 1 mM EDTA, 1 mM EGTA, 1 mM DTT, 1 mM PMSF). The nucleus fraction was collected after removing insoluble membrane debris by centrifugation at 12,000 *g* for 10 min. The NEAT1 and miR-195a expressions in nucleus, cytoplasm, and the whole cell were detected via real-time PCR analysis. The U6 and GAPDH were also used as the internal control of nucleus and cytoplasm, respectively.

### RNA Pull-Down Assay

Briefly, 50 nM biotin-labeled Bio-NC, Bio-miR-195a-WT, or Bio-miR-195a-MUT (RiboBio) were transfected into HNPCs, respectively. After 48 h, the cells were collected and incubated with specific lysis buffer (Thermo Fisher Scientific) to obtain the nucleus and cytoplasmic fractions. The nucleus or cytoplasmic fractions were then incubated with streptavidin magnetic beads (Sigma-Aldrich) at 4°C overnight, respectively. The bounded RNA was eluted and purified for qRT-PCR analysis.

### TUNEL Staining

Briefly, slides (4 μm) were stained with TdT-mediated dUTP nick end labeling (TUNEL) probes (Roche, 11684817910, United States) according to the product instructions. DAPI was used to stain the nuclei. Images were captured using a fluorescence microscope.

### Statistical Analysis

GraphPad Prism 6 software (GraphPad, San Diego, CA, United States) was used for statistical analysis. Data are expressed as mean ± standard deviation. One-way ANOVA was used to perform the comparisons among multiple groups. *P*-value < 0.05 represented that the result was statistically significant.

## Results

### NEAT1 Was Upregulated in IVDD *in vivo* and *in vitro*

**FIGURE 1:**
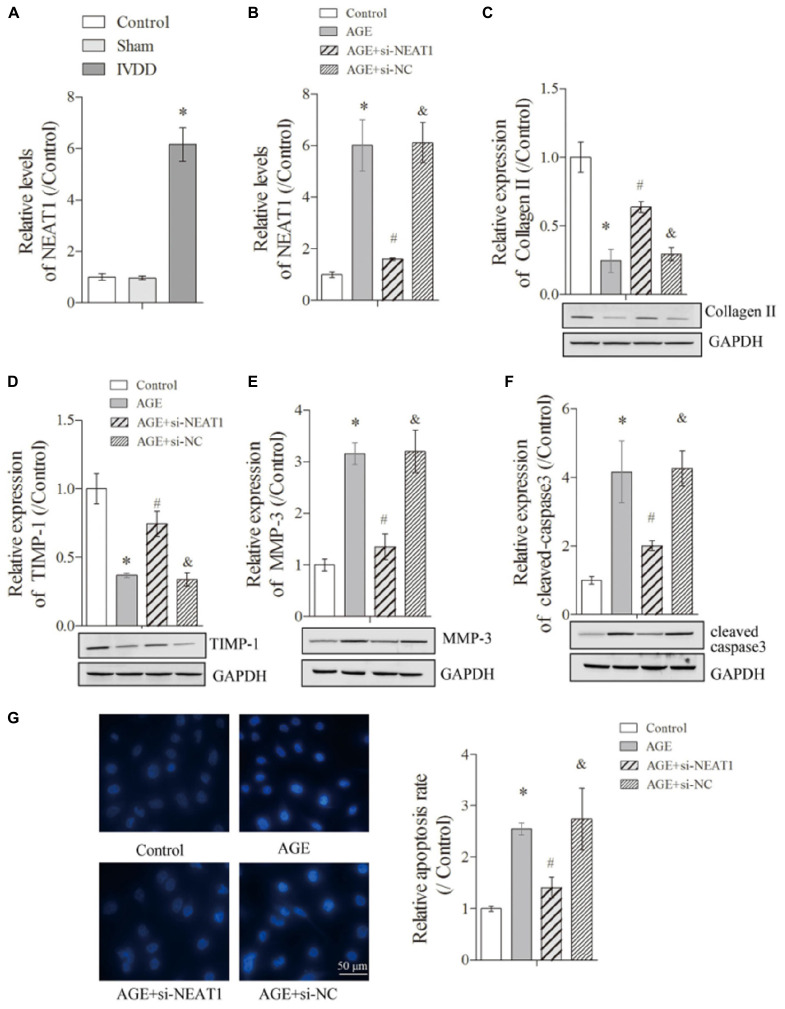
NEAT1 was upregulated in IVDD *in vivo* and *in vitro* and regulated ECM metabolism and apoptosis of degenerative HNPC cells. The expression of NEAT1 *in vivo* **(A)** and AGE or AGE + NEAT1 siRNA-treated HNPC cells was detected by real-time PCR **(B)**; there were three replicates in each group. **(C–F)** The expressions of collagen-II, TIMP-1, MMP-3, and cleaved-caspase-3 were analyzed by Western blot, respectively; there were three replicates in each group. **(G)** Hoechst staining was performed to observe the apoptosis of HNPC cells; there were five replicates in each group. *P < 0.05 *vs*. control group; #*P* < 0.05 *vs*. AGE group; &*P* < 0.05 *vs*. AGE + NEAT1 siRNAs group.

A previous report revealed different expression levels of lncRNA in IVDD by microarray analysis and proved that NEAT1 was highly expressed in IVDD (Lan et al., 2016). Therefore, we selected NEAT1 for further study. To explore the relative expression of NEAT1 in IVDD *in vivo*, we used the AF needle puncture method to construct an IVDD rat model and performed real-time PCR to detect the relative NEAT1 expression. The results showed that NEAT1 was significantly upregulated in the IVDD group compared with the control or sham group (Figure 1A, *P* < 0.05). We further established degenerative HNPC cells by AGE treatment and further knocked out the NEAT1 expression. The results showed that NEAT1 was highly expressed when treated with AGE and greatly suppressed through transfection with NEAT1 siRNAs (Figure 1B, *P* < 0.05).

### NEAT1 Regulated the ECM Metabolism and Apoptosis of Degenerative HNPC Cells

To further detect the influences of NEAT1 on the ECM metabolism in denatured HNPC cells, we first obtained a satisfactory NEAT1 interference efficiency (Figure 1B, *P* < 0.05). The suppression of Collagen-II, a major ECM synthesis protein, by AGE treatment could be reversed under the silencing of NEAT1 (Figure 1C, *P* < 0.05). Besides, we obtained a similar effect of NEAT1 on TIMP-1, which modulated the enzymatic activity of MMP and functioned as a suppressor for ECM-degrading proteins (Figure 1D, *P* < 0.05). However, MMP-3, a major ECM-degrading protein upregulated in degenerative HNPC cells, was largely suppressed after treatment with si-NEAT1 (Figure 1E, *P* < 0.05). More interestingly, the induction of cleaved-caspase-3, the effector of apoptosis, by AGE treatment was also greatly inhibited by silencing of NEAT1 (Figure 1F, *P* < 0.05). Increased apoptosis in degenerative HNPC cells, which was shown in the increased Hoechst nucleus dissolution, was also efficiently abrogated when treated with si-NEAT1 (Figure 1G, *P* < 0.05). All of the above results indicated that upregulated NEAT1 in the IVDD rat model and in degenerative HNPC cells can regulate the ECM metabolism and apoptosis of degenerative HNPC cells.

### NEAT1 Exerted Its Regulatory Role in Degenerative HNPC Cells Partly Through Targeting miR-195a

**FIGURE 2:**
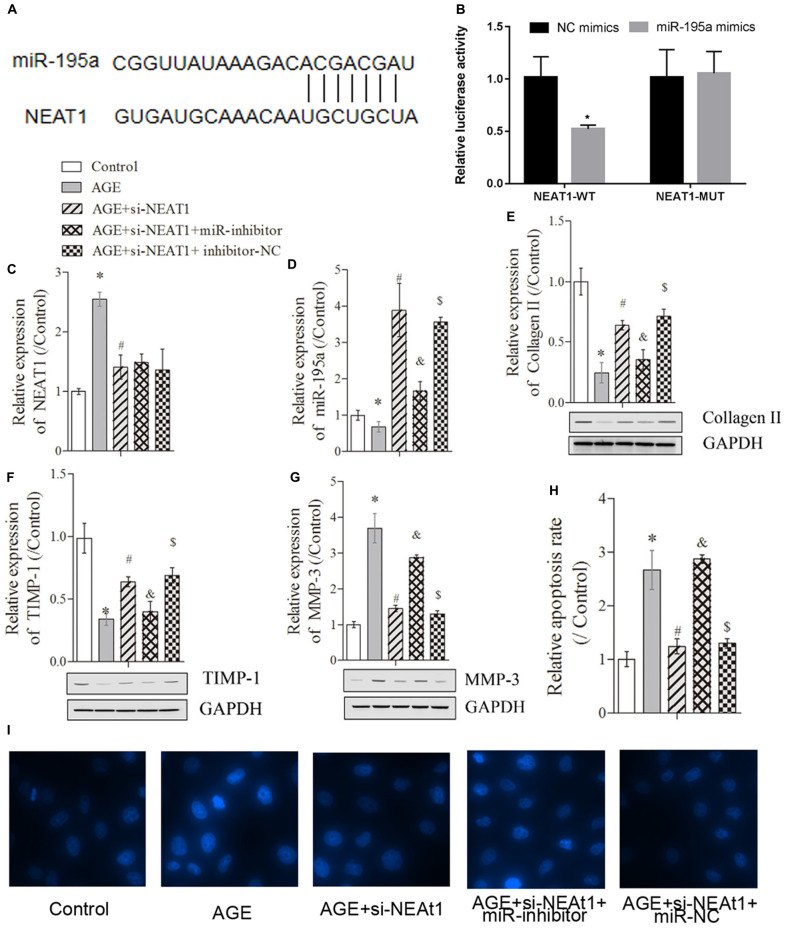
NEAT1 exerted its regulatory role in degenerative HNPC cells through targeting miR-195a. **(A)** The binding sites between miR-195a and NEAT1 were predicted via the StarBase database. **(B)** Luciferase reporter assay was used to explore the relationship between NEAT1 and miR-195a. **(C,D)** Real-time PCR was used to measure the NEAT1 or miR-195a expression in HNPC cells; there were three replicates in each group. **(E–G)** The expressions of collagen-II, TIMP-1, and MMP-3 were analyzed by Western blot, respectively; there were three replicates in each group. **(H,I)** Hoechst staining was performed to observe the apoptosis of HNPC cells; there were five replicates in each group. *P < 0.05 *vs*. control group; #*P* < 0.05 *vs*. AGE group; &*P* < 0.05 *vs*. AGE + NEAT1 siRNAs group; $*P* < 0.05 *vs*. AGE + NEAT1 siRNAs + miR-195a inhibitor group.

To further investigate whether NEAT1 regulated the degenerative HNPC cells through its targeted miRNAs, we used bioinformatics tools (StarBase and miRTarBase) to predict the target miRNA. As shown in Figure 2A, NEAT1 exerted complementary base pairing with miR-195a, which has been reported to regulate deep vein thrombosis via directly targeting Bcl-2 (Jin et al., 2019). To further confirm the interaction between miR-195a and NEAT1, the dual-luciferase reporter system was used to detect the interactions between miR-195a and NEAT1. The luciferase reporter constructs were co-transfected with miR-195a mimics or NC into HEK293 cells. As illustrated in Figure 2B, miR-195a mimics greatly reduced the luciferase activity of NEAT1-WT construct but with no significant changes in NEAT1-MUT construct, indicating a direct interaction between NEAT1 and miR-195a (Figure 2B, *P* < 0.05). Besides, the HNPC cells were transfected with si-NEAT1 and/or miR-195a inhibitors, and the relative expressions of NEAT1 and miR-195a were investigated by real-time PCR. We found that the NEAT1 expression induced by AGE was obviously suppressed by si-NEAT1 transfection. However, there was no significant change in NEAT1 expression after being treated with miR-195a inhibitors or NC (Figure 2C, *P* < 0.05). More interestingly, the relative miR-195a expression, which was significantly suppressed in degenerative HNPC cells, was obviously upregulated after treatment with si-NEAT1 and largely reversed when transfected with miR-195a inhibitors (Figure 2D, *P* < 0.05). Besides, we performed nucleus/cytosolic fraction isolation assay and proved that NEAT1 was mostly located in the nucleus fractions of HNPC cells, while miR-195a was expressed in both the nucleus and cytoplasm (Supplementary Figure S1A). Moreover, the RNA pull-down assay showed that the enrichment of lncRNA NEAT1 was upregulated by the Bio-miR-195a-WT in nucleus fractions but not the cytoplasm when compared with that in the Bio-probe NC group (*P* < 0.05), while the NEAT1 expression exhibited no difference in either nucleus or cytoplasm when treated with Bio-miR-195a-MUT (Supplementary Figure S1B). All of these data confirmed that NEAT1 physically interacted with miR-195a. Proteins that regulate the ECM metabolism were further explored via Western blot assay. Collagen-II (Figure 2E) and TIMP-1 (Figure 2F), which was downregulated in degenerative HNPC cells, was significantly increased by si-NEAT1 treatment. However, the levels of these proteins were largely reversed in the presence of miR-195a inhibitors (*P* < 0.05). For MMP-3, it exhibited the opposite tendency to Collagen-II and TIMP-1 (Figure 2G, *P* < 0.05). Furthermore, Hoechst staining assay indicated that the increased apoptosis in degenerative HNPC cells, which was obviously repressed after silencing of NEAT1, was markedly rescued after being co-transfected with miR-195a inhibitors (Figures 2H,I *P* < 0.05). All of the above results demonstrated that NEAT1 played its regulatory role in degenerative HNPC cells partly via interacting with miR-195a.

### Silencing of NEAT1 Upregulated miR-195a Expression to Reverse the BAX/BAK-Dependent Apoptosis and ECM Metabolism in Degenerative HNPC Cells

**FIGURE 3:**
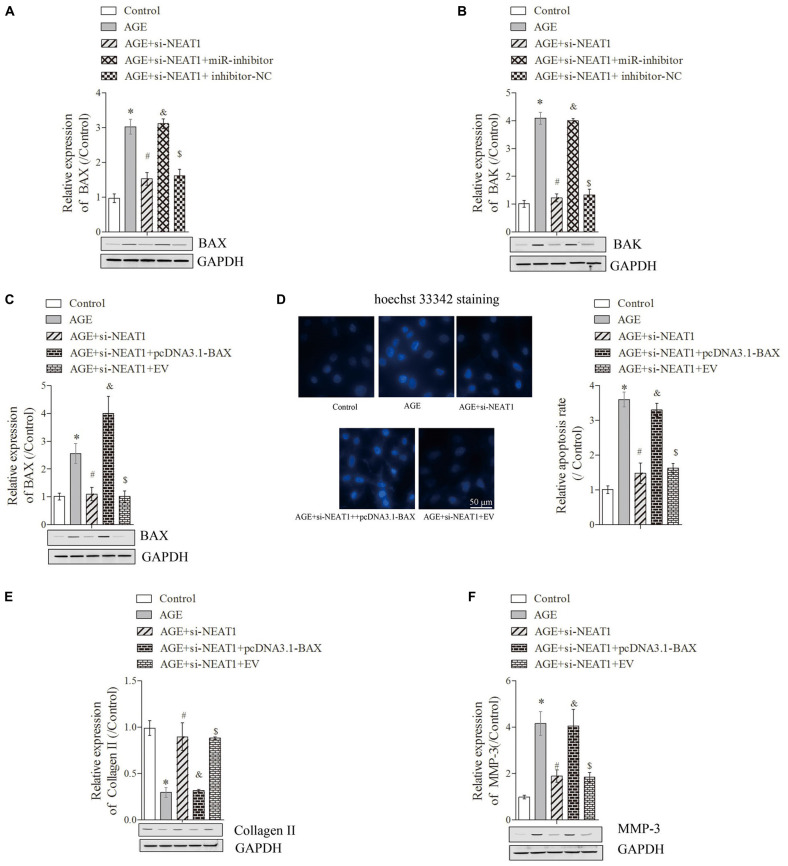
Silencing of NEAT1 upregulates hsa-miR-195a expression to reverse the BAX/BAK-dependent apoptosis and ECM metabolism in degenerative HNPC cells. **(A–C)** The expressions of BAX and BAK were analyzed by Western blot, respectively; there were three replicates in each group. **(D)** Hoechst staining was performed to observe the apoptosis of HNPC cells; there were five replicates in each group. **(E,F)** The expressions of Collagen-II and MMP-3 were measured by Western blot, respectively; there were three replicates in each group. *P < 0.05 *vs*. control group; #*P* < 0.05 *vs*. AGE group; &*P* < 0.05 *vs*. AGE + NEAT1 siRNAs group; $*P* < 0.05 *vs*. AGE + NEAT1 siRNAs + miR-195a inhibitor group.

To detect the related mechanism by which NEAT1 regulates the apoptosis in degenerative HNPC cells, we first used bioinformatics tools (miRTarBase and Informatics) to predict RNA target genes, and the data revealed that Bcl-2 was a target gene of miR-195a (Supplementary Figure S1C). Here, the predicted data and dual-luciferase reporter assay also showed that Bcl-2 could exert complementary base pairing with miR-195a (Supplementary Figure S1D). Given that BAX and BAK are the pro-apoptotic proteins in the Bcl-2 pathway (Edlich, 2018), we selected these proteins to further investigate the changes in key proteins in mitochondrial pathways and demonstrated that the upregulation of pro-apoptotic proteins [BAX (Figure 3A) and BAK (Figure 3B)] induced by AGE was obviously suppressed by si-NEAT1 treatment but that these effects were largely rescued when co-transfected with miR-195a inhibitors (*P* < 0.05). To further confirm whether NEAT1 regulates the apoptosis of degenerative HNPC cells via mediating the BAX/BAK pathway, si-NEAT1 and miR-195a inhibitors were co-transfected into HNPC cells, and the transfection efficiency was explored by Western blot. Results showed that the BAX expression suppressed by si-NEAT1 in degenerative HNPC cells was significantly upregulated after transfection with pcDNA3.1-BAX plasmids (Figure 3C, *P* < 0.05). We further investigated whether NEAT1 affects the apoptosis of degenerative HNPC cells through regulating BAX. As shown in Figure 3D, the apoptosis of degenerative HNPC cells repressed by NEAT1 silencing was obviously rescued after being transfected with pcDNA3.1-BAX plasmids, further indicating that NEAT1 regulated the apoptosis in degenerative HNPC cells via mediating the BAX/BAK pathway (Figure 3D, *P* < 0.05). The impact of NEAT1 on Collagen II expression under BAX overexpression was also explored. Results were obtained that showed that the upregulation of Collagen II expression by NEAT1 knockdown was dramatically decreased after co-transfection with si-NEAT1 and pcDNA3.1-BAX plasmids (Figure 3E, *P* < 0.05). For MMP-3, however, there was the opposite trend to that of Collagen II expression (Figure 3F, *P* < 0.05). These results indicated that NEAT1 may mediate the ECM metabolism in the degeneration of HNPC cells through regulating the BAX/BAK pathway.

### Silencing of NEAT1 Upregulated miR-195a to Resist BAX/BAK-Dependent Apoptosis and ECM Metabolism in the IVDD Rat Model

**FIGURE 4:**
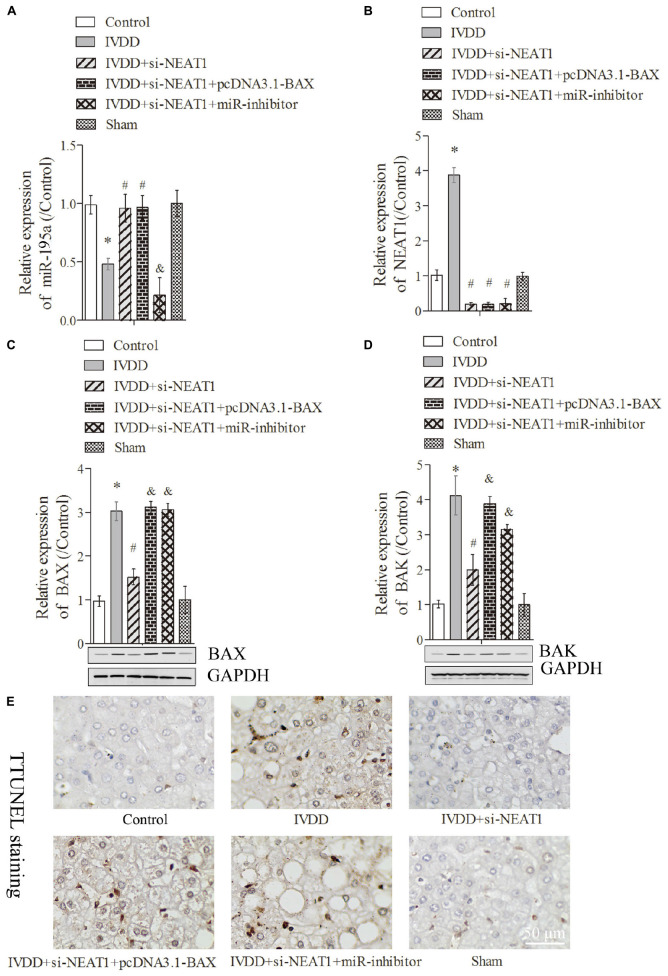
Silencing of NEAT1 upregulates miR-195a to reverse BAX/BAK-dependent apoptosis in the IVDD rat model. **(A,B)** Real-time PCR was used to measure the miR-195a or NEAT1 expression in the IVDD rat model; there were three replicates in each group. **(C,D)** The expressions of BAX and BAK were analyzed by Western blot, respectively; there were three replicates in each group. **(E)** Tunel staining was used to detect the apoptosis of nucleus pulposus cells in the IVDD rat model. *P < 0.05 *vs*. control group; #*P* < 0.05 *vs*. IVDD group; &*P* < 0.05 *vs*. IVDD + NEAT1 siRNAs group.

**FIGURE 5:**
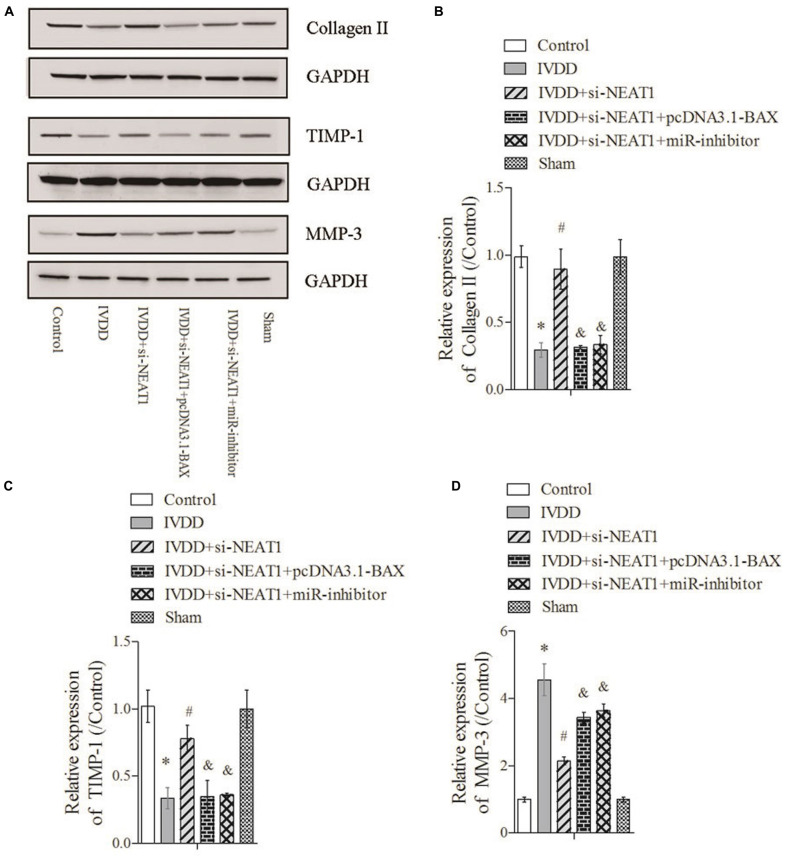
Silencing of NEAT1 upregulates miR-195a to reverse BAX/BAK-dependent ECM metabolism in the IVDD rat model. **(A)** The expressions of Collagen-II, TIMP-1, and MMP-3 were analyzed by Western blot, respectively. **(B–D)** Semi-quantitative analysis of proteins; there were three replicates in each group. *P < 0.05 *vs*. control group; #*P* < 0.05 *vs*. IVDD group; &*P* < 0.05 *vs*. IVDD + NEAT1 siRNAs group.

The rat IVDD model was constructed and used to observe the effect of NEAT1 and miR-195a on apoptosis *in vivo*. Real-time PCR was performed to detect the relative expressions of miR-195a and NEAT1, respectively. The results showed that miR-195a was significantly downregulated in the IVDD group compared to the control or sham group (Figure 4A, *P* < 0.05). Moreover, the downregulated relative miR-195a expression in the IVDD group was obviously increased after transfection with NEAT1 siRNAs alone or co-transfection with pcDNA3.1-BAX plasmids (Figure 4A, *P* < 0.05). Furthermore, co-transfection of NEAT1 siRNAs and miR-195a inhibitors significantly downregulated the expression of miR-195a when compared to the NEAT1 siRNA group in the IVDD model (Figure 4A, *P* < 0.05). The expression of NEAT1 was significantly decreased after transfection with NEAT1 siRNAs alone or co-transfected with pcDNA3.1-BAX plasmids or miR-195a inhibitors in the IVDD model (Figure 4B, *P* < 0.05). We also demonstrated that si-NEAT1 treatment obviously repressed the increase in BAX (Figure 4C) and BAK expression (Figure 4D) in the IVDD model but that these effects were largely rescued when co-transfected with si-NEAT1 and pcDNA3.1-BAX plasmids or miR-195a inhibitors, respectively (*P* < 0.05). In addition, TUNEL staining was used to detect apoptosis in nucleus pulposus. The data indicated that the increased apoptosis in the IVDD model, which was obviously repressed after silencing of NEAT1, was markedly rescued after being co-transfected with pcDNA3.1-BAX plasmids or miR-195a inhibitors (Figure 4E, *P* < 0.05). Proteins that regulate ECM metabolism were further explored via Western blot assay. Collagen-II (Figures 5A,B) and TIMP-1 (Figures 5A,C), which were downregulated in the IVDD model, were significantly increased by si-NEAT1 treatment. However, the levels of these proteins were largely reversed in the presence of pcDNA3.1-BAX plasmids or miR-195a inhibitors (*P* < 0.05). MMP-3, however, showed opposite results to those for Collagen-II and TIMP-1 (Figures 5A,D, *P* < 0.05). All of the above data indicated that silencing of NEAT1 upregulates miR-195a to resist BAX/BAK-dependent apoptosis and ECM metabolism in the IVDD rat model.

## Discussion

Recently, researchers have proved that there is an interactive regulatory network between lncRNA and miRNA. LncRNA can compete with or block the function of miRNA to relieve the regulation of miRNA on mRNA (Xia et al., 2014). Meanwhile, miRNA can bind to lncRNA with the help of other RNA binding proteins, and activation of miRNA-mediated degradation or silencing mechanisms can adjust the stability of lncRNA (Li N. et al., 2017). Wang et al. (2018) found that lncRNA H19 can regulate the H_2_O_2_-mediated deregulation in NP cell senescence and proliferation through targeting miR-22. Wang et al. (2017) have proved that lncRNA-RMRP can accelerate the proliferation of NP cells by mediating the miR-206 expression. Furthermore, Yan et al. (2019) showed that NEAT1 functions as a sponge of miR-125a-5p to suppress cardiomyocyte apoptosis. Chen J.X. et al. (2019) also demonstrated that NEAT1 can repress immunity through accelerating the reduction of miR-125-dependent MCEMP1. In this study, we proved that NEAT1 regulated the metabolism imbalance of ECM and the apoptosis of NP cells through interacting with miR-195a. Although we predicted that there were many miRNAs including miR-195a binding with NEAT1 via the bioinformatics tool (StarBase), we further predicted the regulatory interaction between miR-195a and the downstream genes using miRTarBase and Informatics to explore the miRNA that might participate in the regulation of IVDD. A report has demonstrated that Bcl-2 is a target gene of miR-195a (Jin et al., 2019), and other reports have proven that Bcl-2 regulates mitochondria-initiated apoptosis. Given that the reduction of NP cells caused by apoptosis is one of the main morphological changes of IVDD, we selected miR-195a for further study. However, whether the instability of lncRNA could be influenced by miR-195a requires further study.

The reduction of NP cells caused by apoptosis is one of the main morphological changes in IVDD. The mitochondrial pathways, death receptor pathway, and endoplasmic reticulum stress pathway are the three major signal pathways for intervertebral disk apoptosis and participate in apoptosis at different stages of IVDD (Wang et al., 2011). Mitochondrial pathways play a major role in moderate to severe degeneration. In this signaling pathway, the release of *cytochrome c* (*Cyt-c*) from mitochondria is promoted by the downregulation of Bcl-2 (an anti-apoptotic protein) and upregulation of Bax/Bak (pro-apoptotic proteins) (Ott et al., 2007; Vince et al., 2018). The released *Cyt-c* then combines with pro-caspase-9 to form an apoptosome that further activates caspase-3 to trigger a cascade of caspases, which finally leads to apoptosis and accelerates the disk degeneration (Ding et al., 2012). Here, we found that NEAT1 can activate the mitochondrial apoptosis pathway in NP cells through upregulating BAX/BAK proteins and cleaved caspase-3 protein to promote apoptosis. More interestingly, the effect of NEAT1 on BAX/BAK proteins was largely reversed by miR-195a. These findings revealed that the upregulated NEAT1 in NP cells can bind directly to miR-195a to promote the activation of the mitochondrial pathway, thereby inducing apoptosis of NP cells. Many reports have proved that lncRNAs mediate the expression of miRNAs through epigenetic regulation (Li M.A. et al., 2017; Cheng et al., 2018). Cheng et al. (2018) proved that HOTAIR-induced epigenetic suppression of miR-122 via DNA methylation was mediated by DNA methyltransferases (DNMTs) that catalyze the DNA methylation. Given that we also proved that both NEAT1 and part of miR-195a existed in nucleus, we hypothesized that NEAT1 might regulate the miR-195a via DNA methylation. However, we still need to perform further experiments to demonstrate whether the NEAT1-induced miR-195a suppression was associated with enhanced DNMT expression or whether this effect was abolished by inhibiting DNMT expression.

Many researchers have proved that an imbalance of ECM synthesis and degradation is the main reason for the pathological progression of IVDD (Liu et al., 2018). Interestingly, this imbalance of metabolism is regulated by a series of cytokines including positive regulators such as transforming growth factor-β (TGF-β), bone morphogenetic proteins (BMPs) that promote the proliferation of intervertebral disk cells and the synthesis of ECM, and inverse regulators such as MMP-1 and MMP-13 that can accelerate the decomposition of matrix (Peeters et al., 2015; Chen S. et al., 2019; Shi et al., 2019; Wang et al., 2019). Besides, these positive regulators and tissue inhibitor of metalloproteinases (TIMPs) promote the synthesis of ECM to prevent, delay, or reverse IVDD by increasing the Aggrecan and Collagen II gene expression (Liu et al., 2017; Zhang et al., 2018). Here, we demonstrated that downregulation of NEAT1 significantly suppressed MMP-3 expression and promoted TIMP-1 expression, indicating that NEAT1 might play a regulatory role in IVDD through mediating the synthesis and degradation of ECM. Moreover, researchers have also demonstrated that the biological effects of regulators that control the balance of ECM synthesis and degradation are often limited in short half-lives (Birch, 2018). Therefore, we will further conduct experiments to explore whether NEAT1 can continuously express or autonomously replenish specific cytokines or regulators by activating degenerative disk cells.

Autophagy is an important way to regulate ECM metabolism and cell apoptosis of intervertebral disk. Jiang et al. (2014) found that the number of autophagosomes is significantly decreased in NP cells of patients with IVDD; meanwhile, the LC3-II/LC3-I ratio and Beclin 1 expression level are also decreased, but the autophagy inhibitors 3- methyl adenine can significantly reduce the number of autophagic bubbles (Ye et al., 2013). Interestingly, studies have proved that pro-inflammatory cytokines, including TNF-α and interleukin-1β (IL-1β), are upregulated in IVDD and promote the levels of MMPs and ADAMTSs (Johnson et al., 2015). Wang et al. (2016) found in nucleus pulposus cells that resveratrol (RSV) can protect NP cells by activating autophagy to suppress TNF-α-induced MMP-3 expression. More interestingly, as an independent type II programmed cell death process, autophagy is closely associated with apoptosis. Jiang et al. (2014) found that silencing of information regulation 2 homolog 1 (SIRT1) can inhibit apoptosis of NP cells by upregulating autophagy. It seems that autophagy helps prevent the degradation of ECM under inflammatory conditions and suppresses apoptosis of NP cells, thereby protecting against IVDD. Hence, we will further explore the role of NEAT1 in regulating autophagy during IVDD.

**FIGURE 6:**
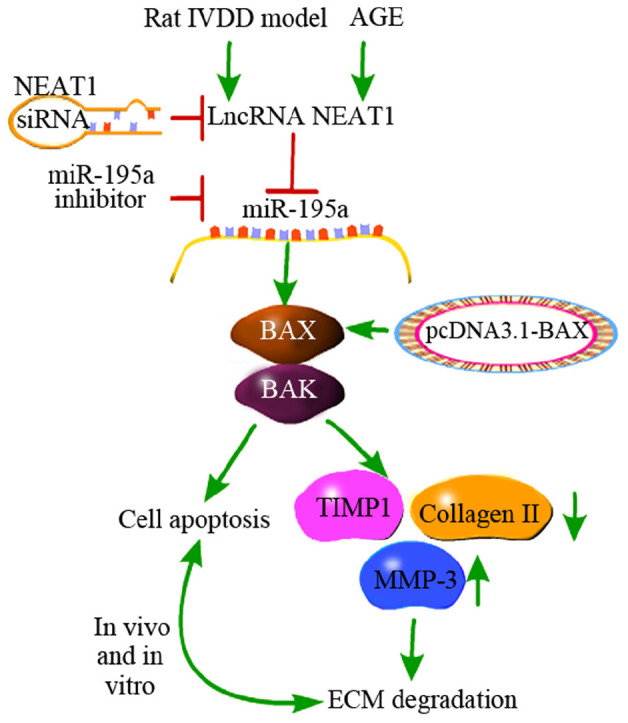
A schematic diagram depicts the molecular basis by which lncRNA NEAT1 downregulates miR-195a to promote BAX/BAK-dependent apoptosis and extracellular matrix degradation *in vitro* and *in vivo.* Downregulation of NEAT1 significantly suppressed the apoptosis induced by AGE in nucleus pulposus cells, while silencing of miR-195a or over-expression of BAX obviously reversed this effect. Moreover, negative regulation of miR-195a by NEAT1 resulted in upregulation of MMP-3, cleaved caspase-3, BAX, and BAK and downregulation of Collagen II and TIMP-1, which are associated with EMT and apoptosis.

In conclusion, we noted that NEAT1 was upregulated in IVDD *in vivo* and *in vitro*. Besides, we first proved that downregulation of NEAT1 significantly suppressed the apoptosis induced by AGE in NP cells, whereas silencing of miR-195a or overexpression of BAX obviously reversed this effect. Mechanically, NEAT1 competed with miR-195a, resulting in upregulation of MMP-3, cleaved caspase-3, BAX, and BAK, as well as downregulation of Collagen II and TIMP-1, which are associated with EMT and apoptosis (Figure 6). This study suggests that both NEAT1 and miR-195a may be potential novel targets for the treatment of IVDD.

## Data Availability Statement

The datasets presented in this study can be found in online repositories. The names of the repositories and accession numbers can be found in the article/Supplementary Material.

## Ethics Statement

The animal study was reviewed and approved by the Ethics Committee on Animal Care and Use of Chinese Academy of Medical Sciences Peking Union Medical College Hospital.

## Author Contributions

NT and YD performed the experiment. JL performed the data analysis. HZ designed the study. NT prepared the manuscript. All authors contributed to the article and approved the submitted version.

## Conflict of Interest

The authors declare that the research was conducted in the absence of any commercial or financial relationships that could be construed as a potential conflict of interest.

## Supplementary Material

FIGURE S1 The results of nucleus/cytosolic fractionation and miR-195a target gene prediction. **(A)** The relative expressions of NEAT1 and miR-195a in nucleus/cytosolic fractionation were detected via real-time PCR; there were three replicates in each group. **P* < 0.05 *vs*. Whole cell. **(B)** RNA pull-down assay was performed to validate the interaction between NEAT1 and miR-195a. **P* < 0.05 *vs*. Bio-probe NC. **(C)** The target genes of miR-195a were predicted by bioinformatics tools (miRTarBase and Informatics). **(D)** The binding sites between miR-195a and Bcl-2; the luciferase reporter assay was used to explore the relationship between miR-195a and Bcl-2. The “*” represents *P* < 0.05 in the comparison between miR-195a mimics and NC mimics.
