# Supplementary figures and images for: Silencing of Long Non-coding RNA NEAT1 Upregulates miR-195a to Attenuate Intervertebral Disk Degeneration via the BAX/BAK Pathway
Source: Front Mol Biosci. 2020 Aug 11;7:147. doi: 10.3389/fmolb.2020.00147 (PMC7433405; doi:10.3389/fmolb.2020.00147)

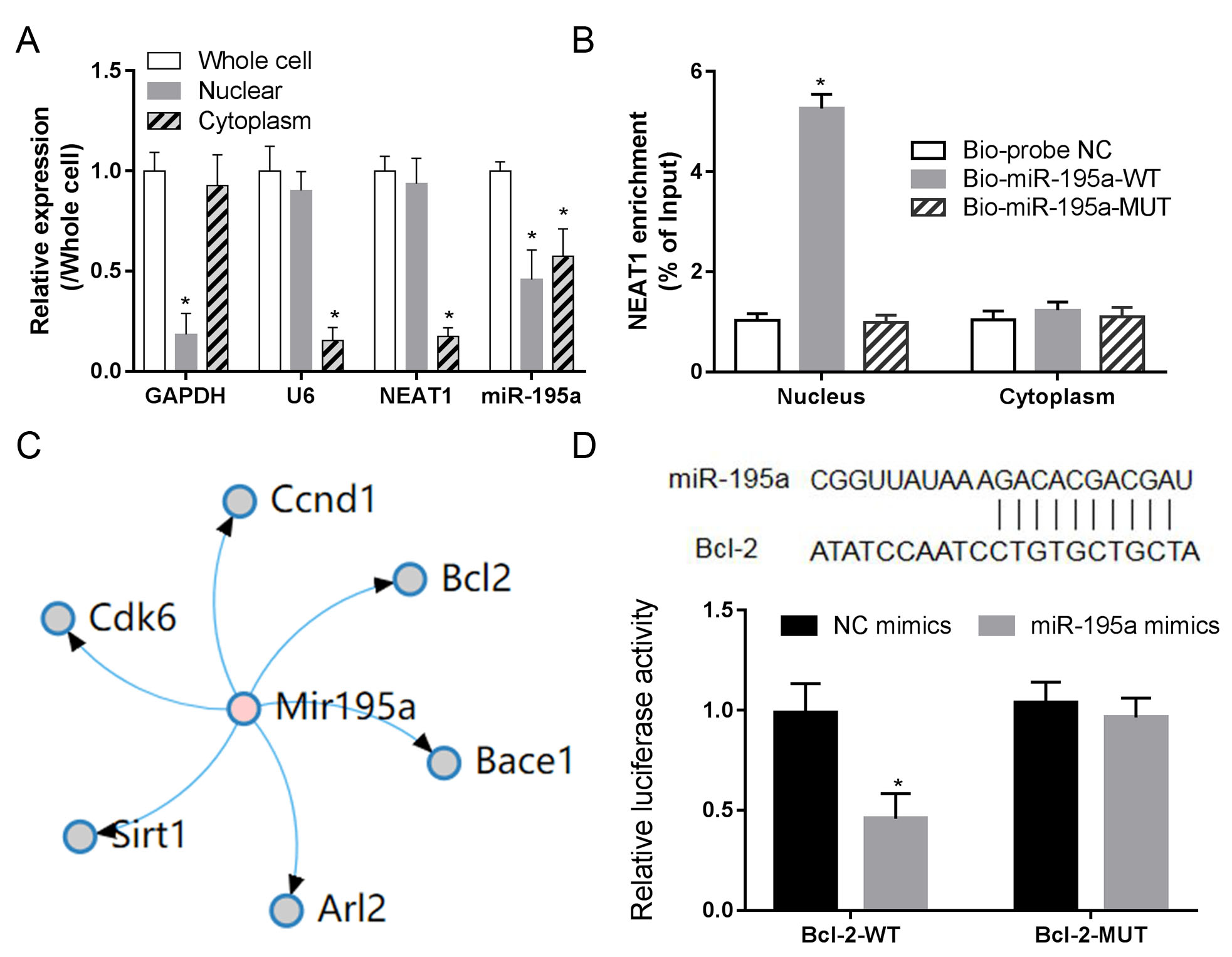

Supplement: FIGURE S1 — The results of nucleus/cytosolic fractionation and miR-195a target gene prediction. (A) The relative expressions of NEAT1 and miR-195a in nucleus/cytosolic fractionation were detected via real-time PCR; there were three replicates in each group. *P < 0.05 vs. Whole cell. (B) RNA pull-down assay was performed to validate the interaction between NEAT1 and miR-195a. *P < 0.05 vs. Bio-probe NC. (C) The target genes of miR-195a were predicted by bioinformatics tools (miRTarBase and Informatics). (D) The binding sites between miR-195a and Bcl-2; the luciferase reporter assay was used to explore the relationship between miR-195a and Bcl-2. The “*” represents P < 0.05 in the comparison between miR-195a mimics and NC mimics. [file Image_1.jpeg]

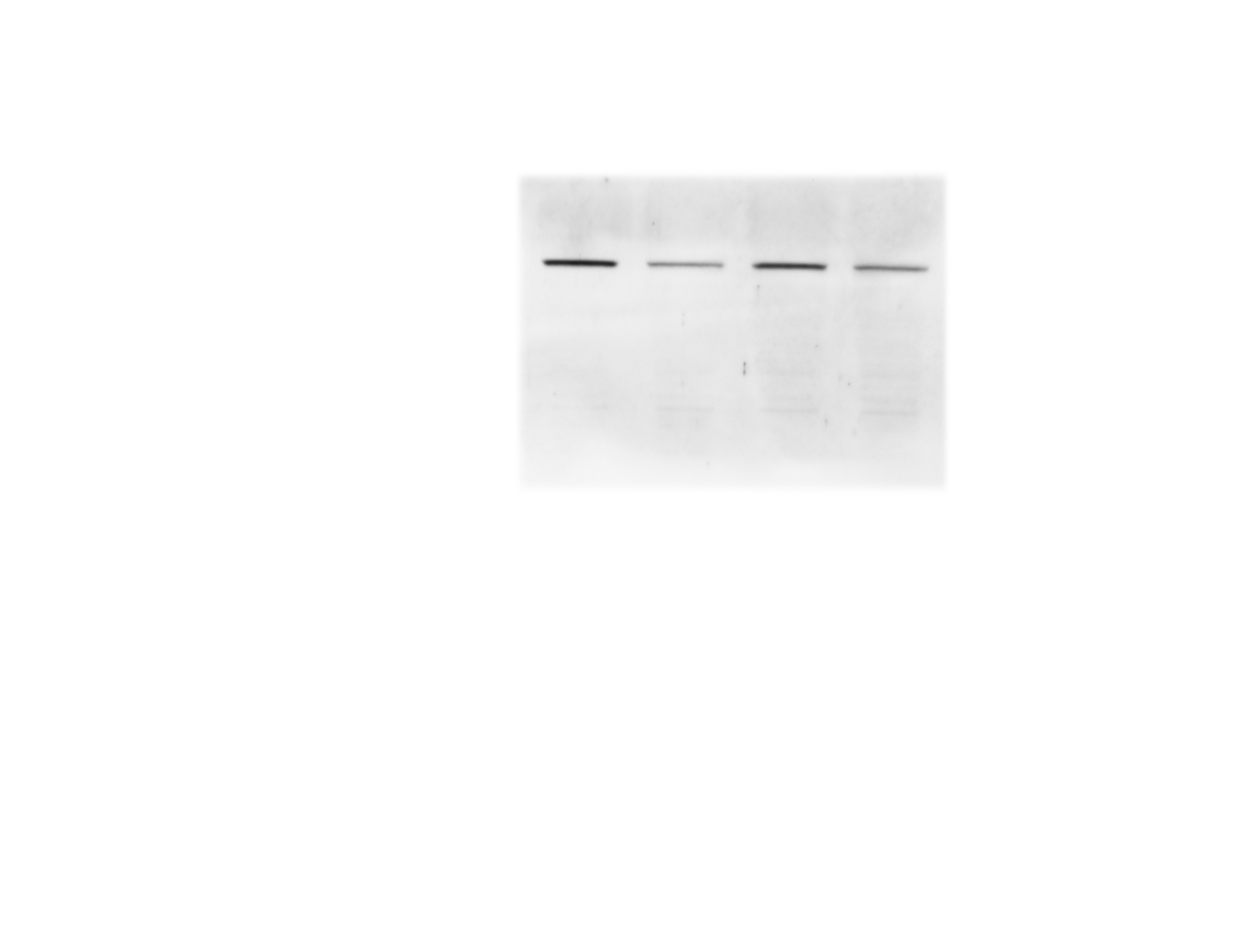

Supplement: Supplementary file 2 [file Data_Sheet_1.ZIP › Original WB images/FIG1/Collagen II.tif]

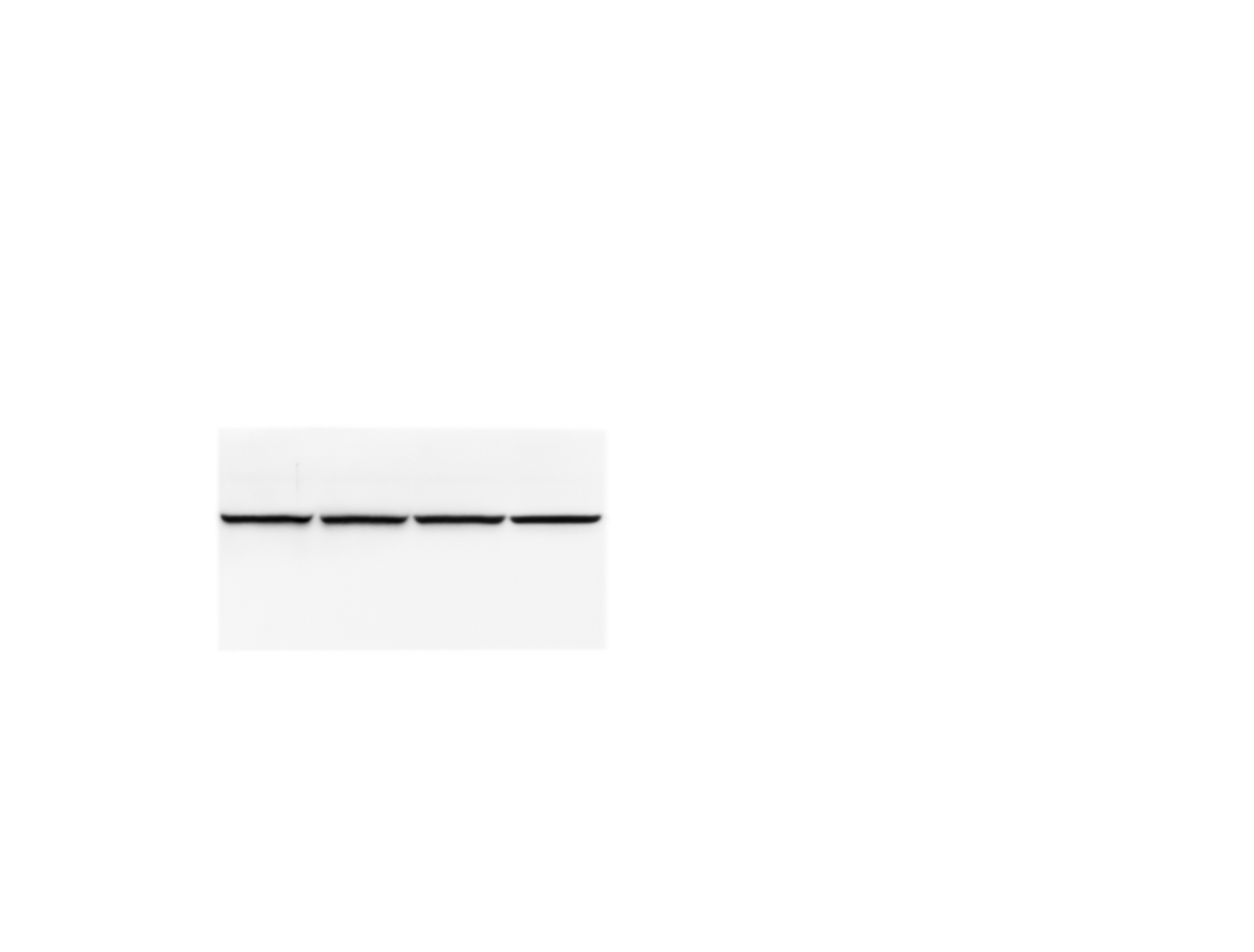

Supplement: Supplementary file 2 [file Data_Sheet_1.ZIP › Original WB images/FIG1/GAPDH 1.tif]

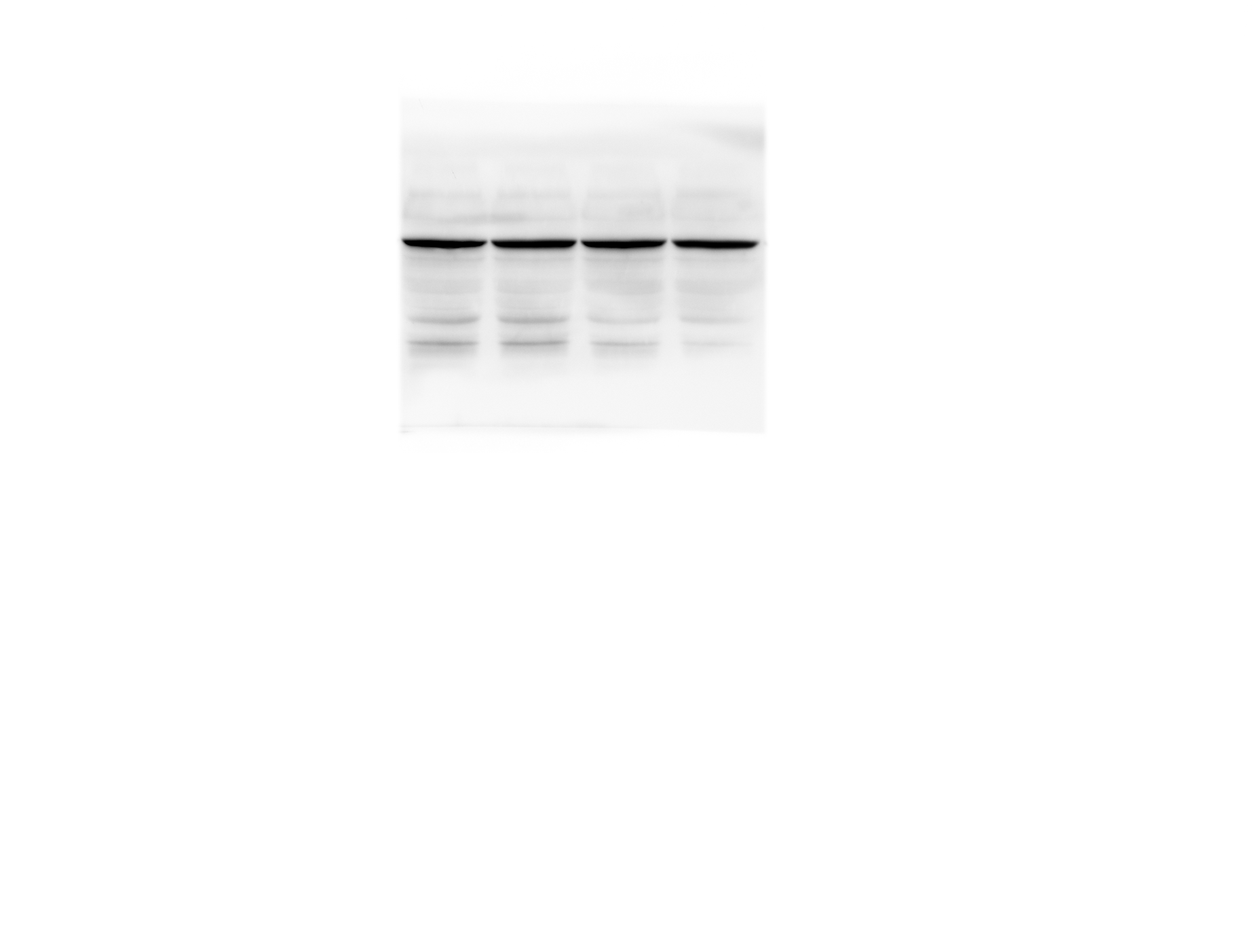

Supplement: Supplementary file 2 [file Data_Sheet_1.ZIP › Original WB images/FIG1/GAPDH 2.tif]

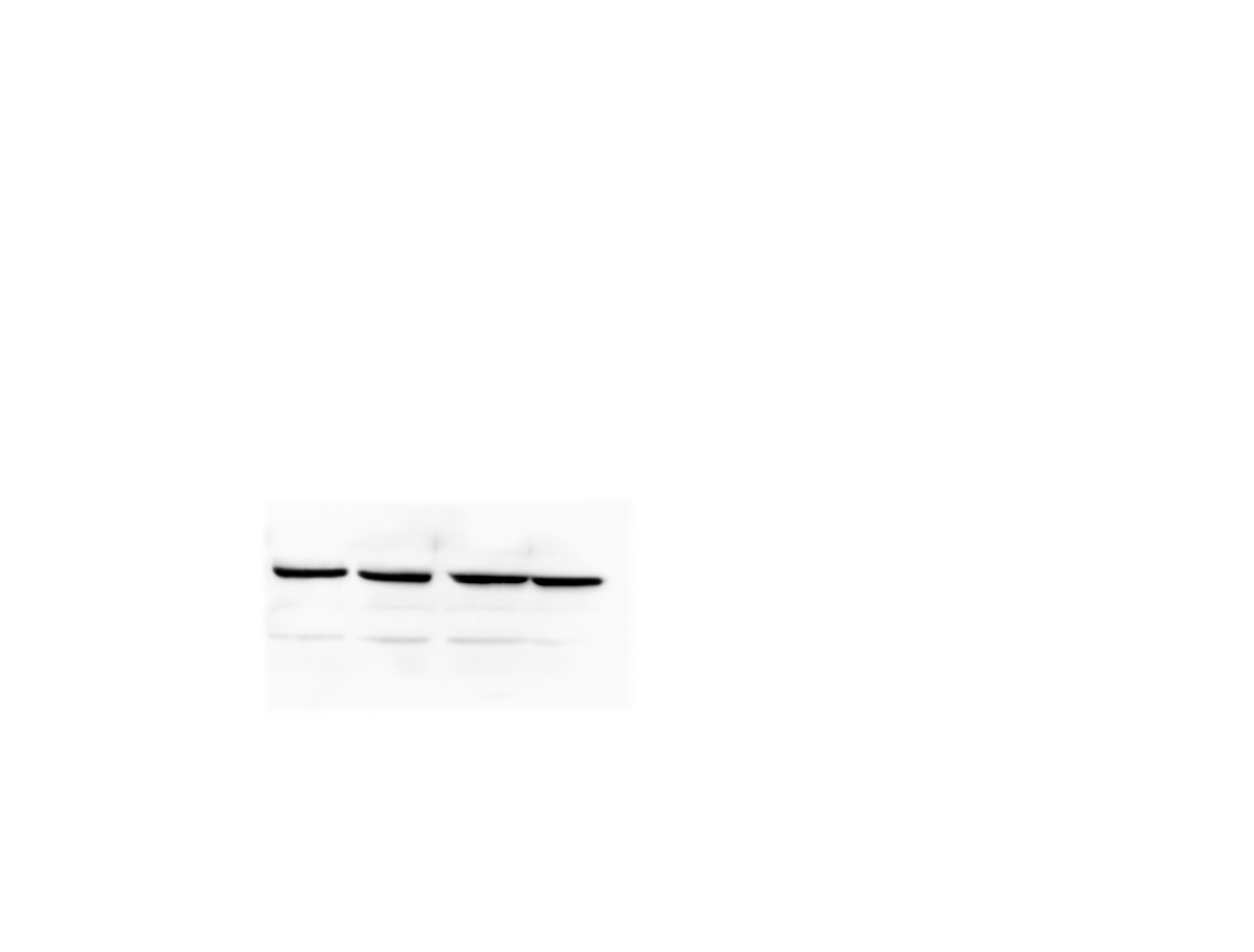

Supplement: Supplementary file 2 [file Data_Sheet_1.ZIP › Original WB images/FIG1/GAPDH 3.tif]

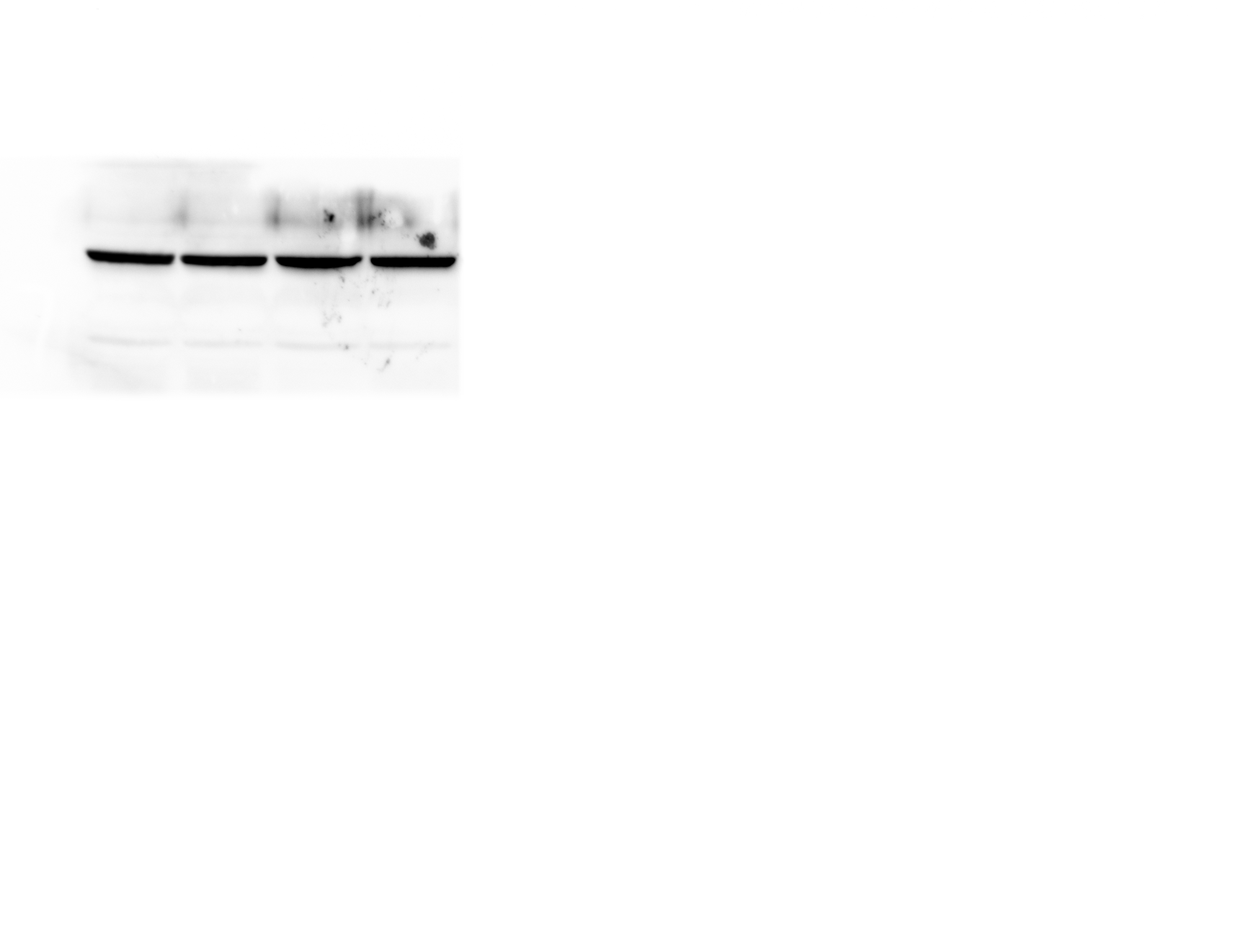

Supplement: Supplementary file 2 [file Data_Sheet_1.ZIP › Original WB images/FIG1/GAPDH 4.tif]

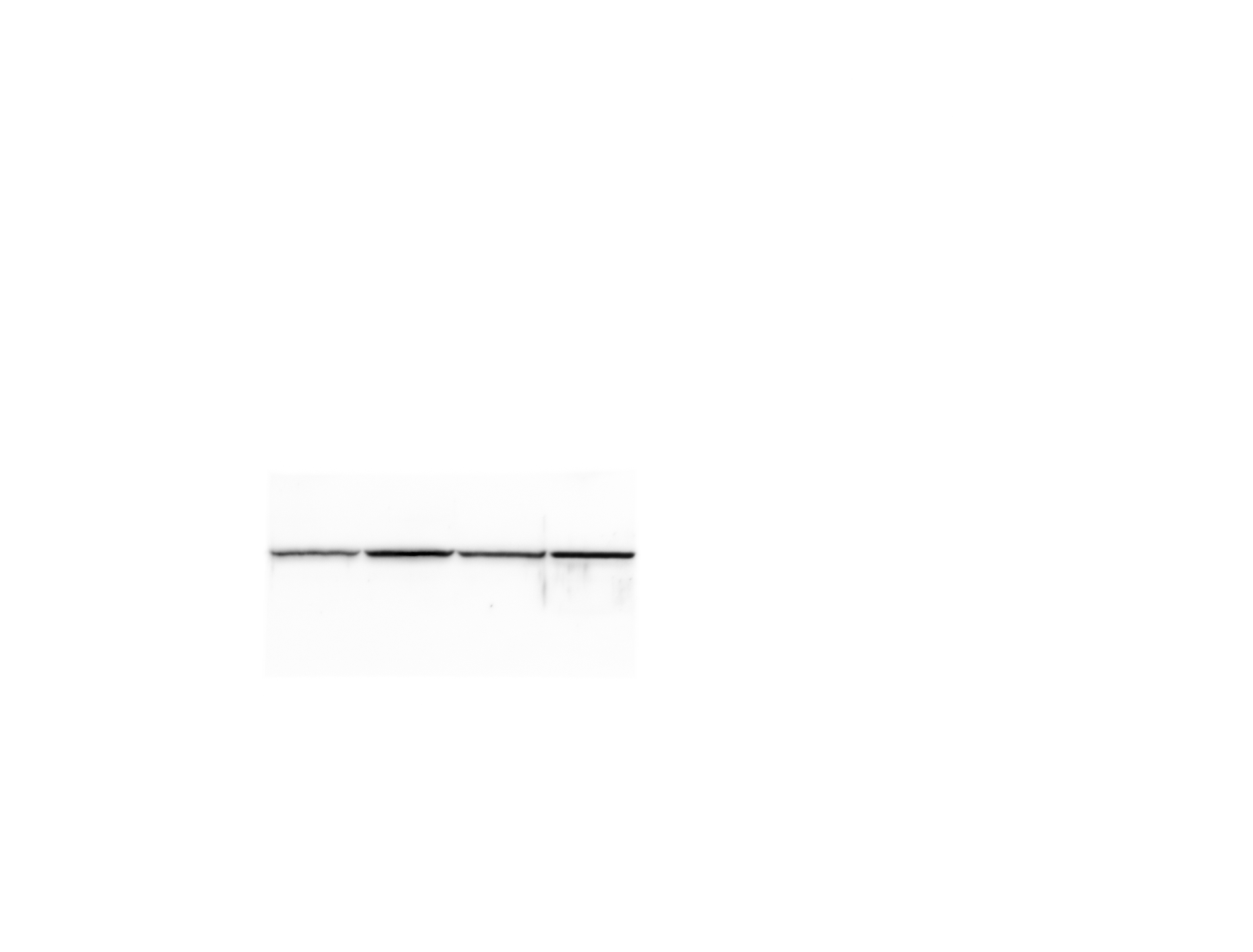

Supplement: Supplementary file 2 [file Data_Sheet_1.ZIP › Original WB images/FIG1/MMP-3.tif]

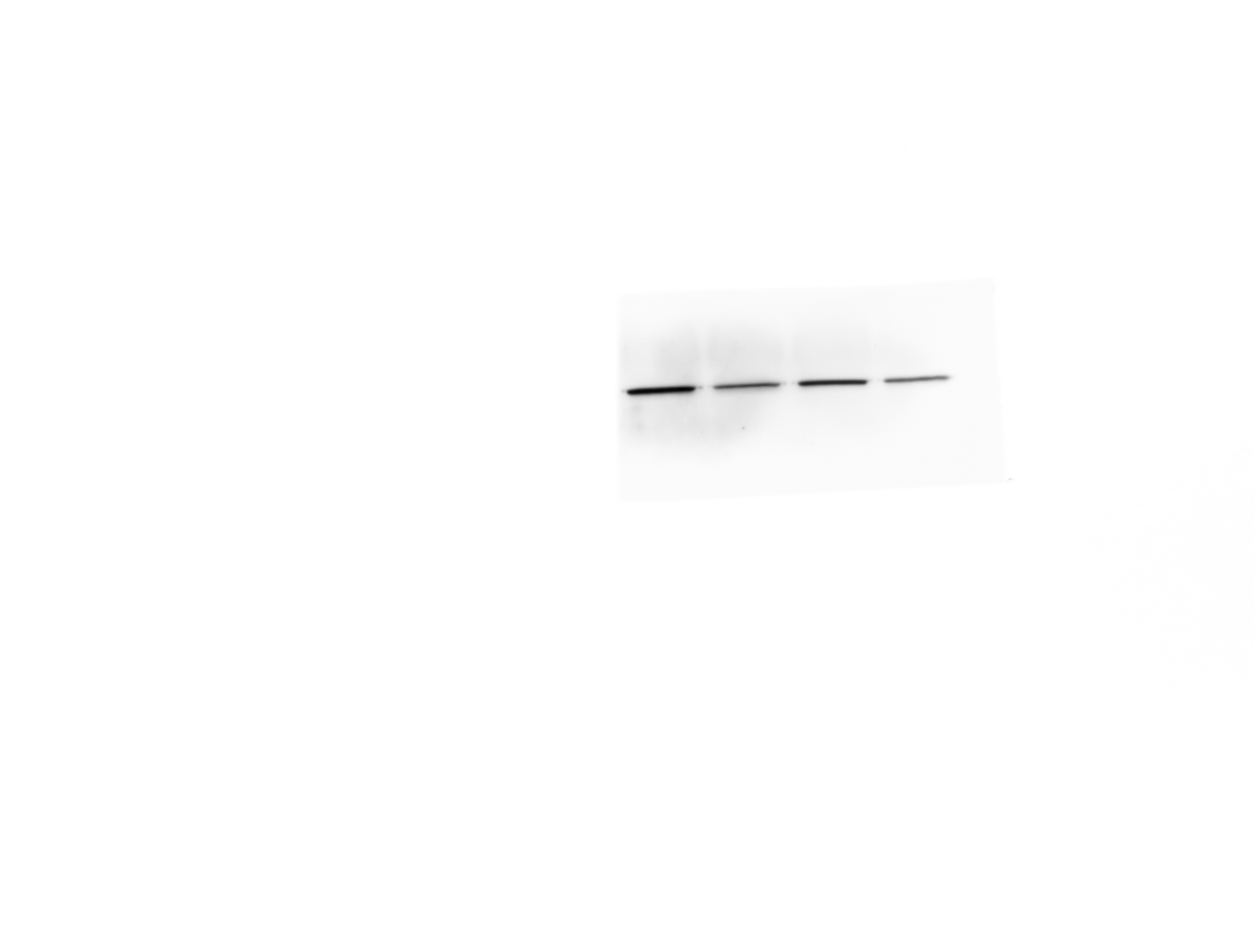

Supplement: Supplementary file 2 [file Data_Sheet_1.ZIP › Original WB images/FIG1/TIMP-1.tif]

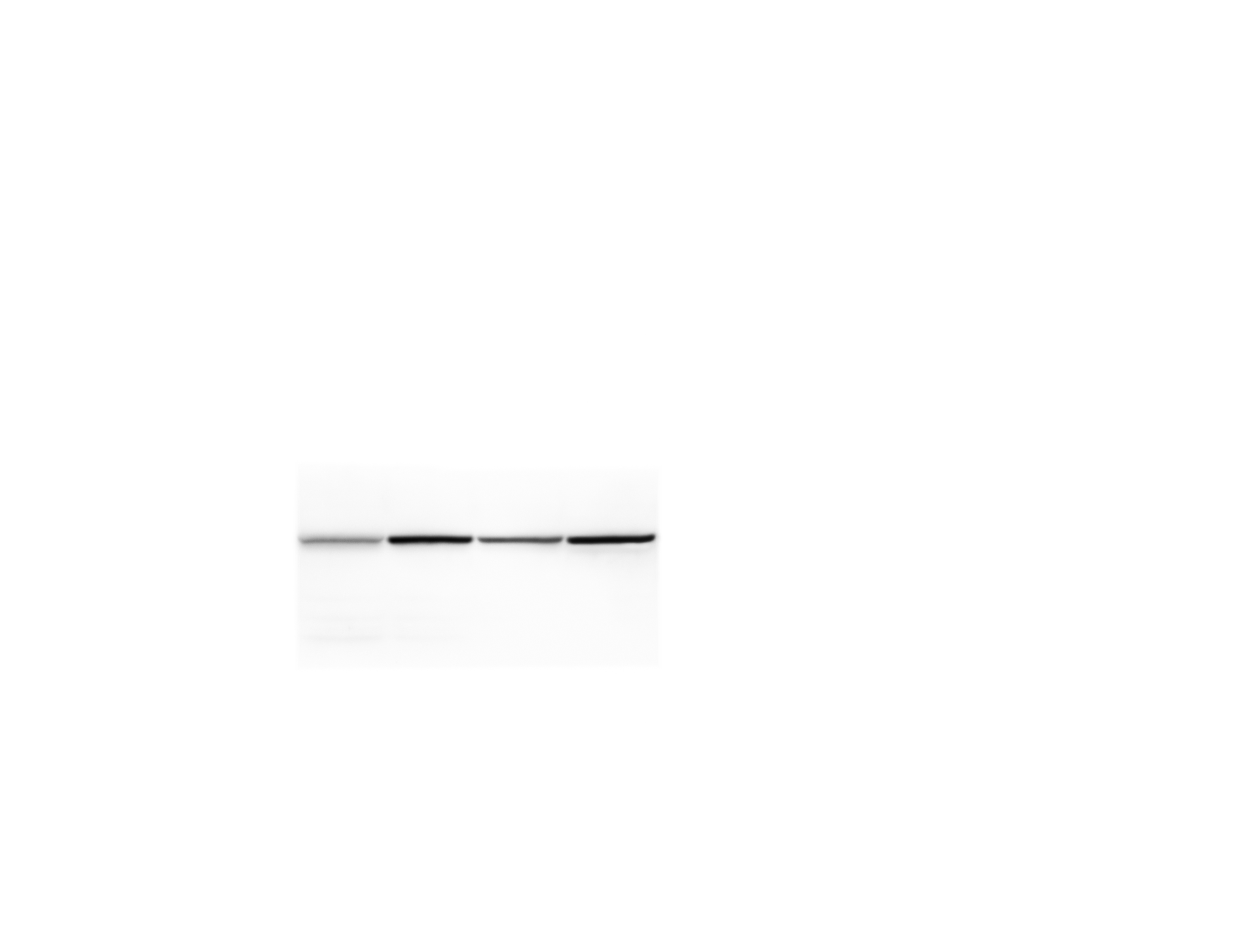

Supplement: Supplementary file 2 [file Data_Sheet_1.ZIP › Original WB images/FIG1/cleaved-caspase3.tif]

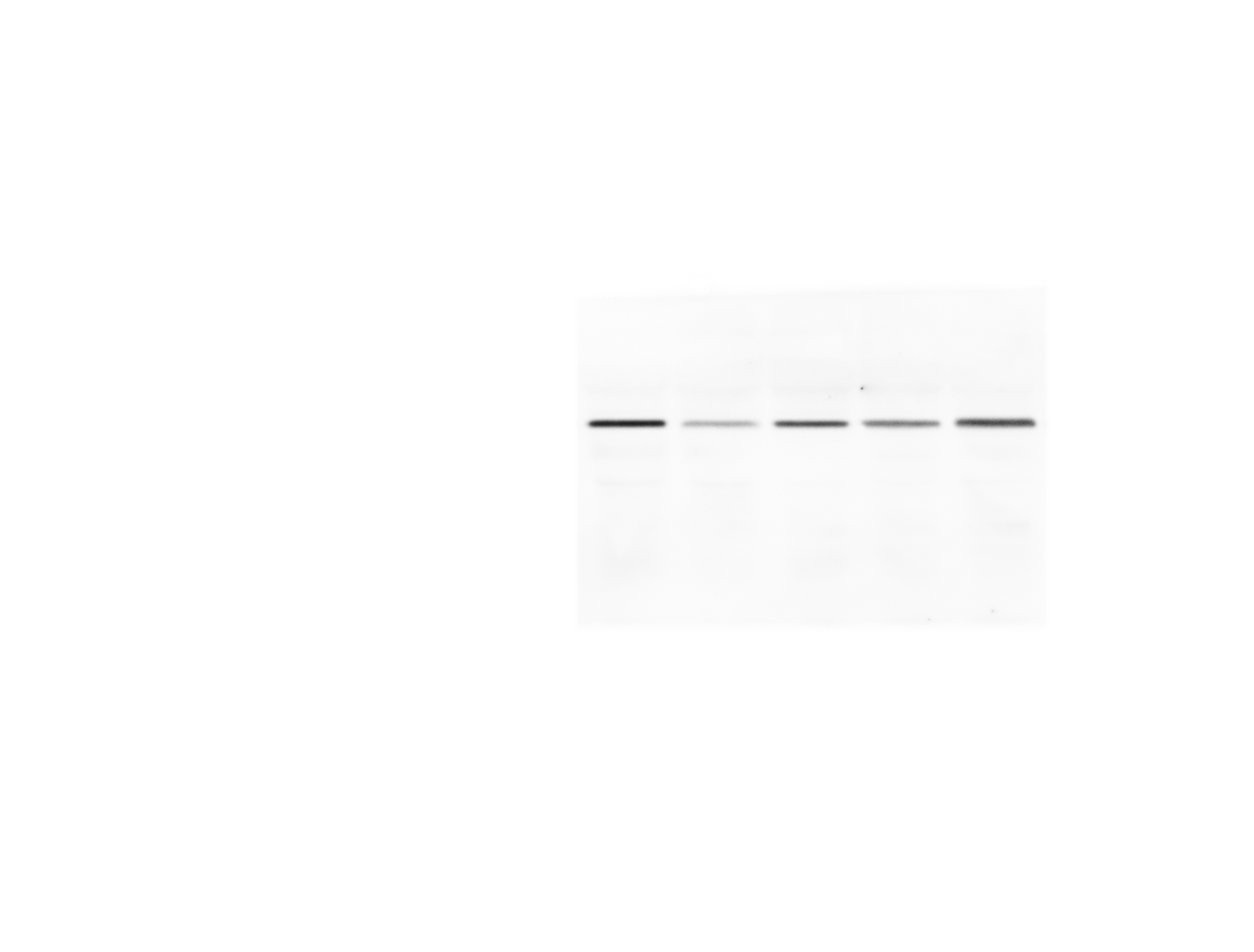

Supplement: Supplementary file 2 [file Data_Sheet_1.ZIP › Original WB images/FIG2/D/Collagen II.tif]

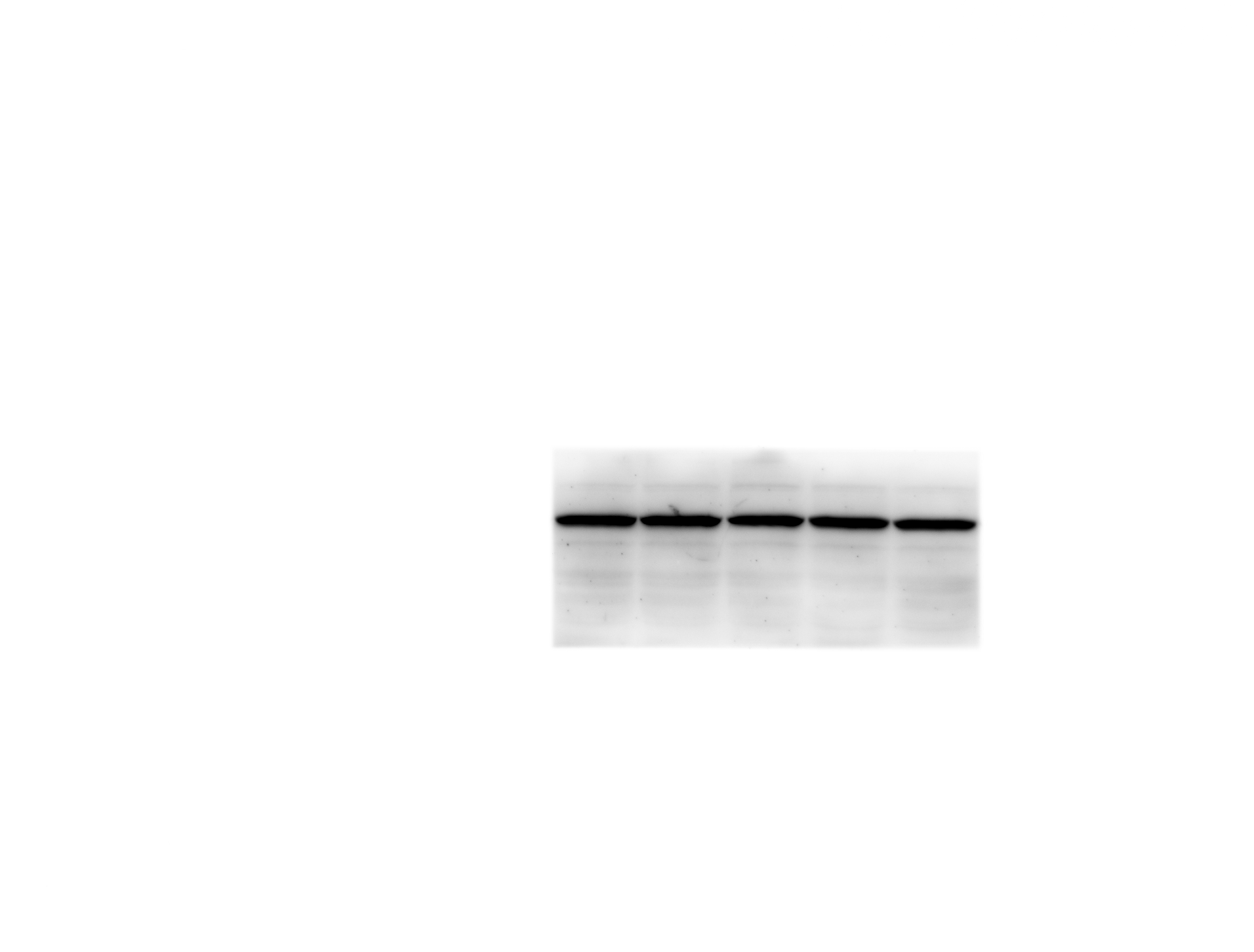

Supplement: Supplementary file 2 [file Data_Sheet_1.ZIP › Original WB images/FIG2/D/GAPDH.tif]

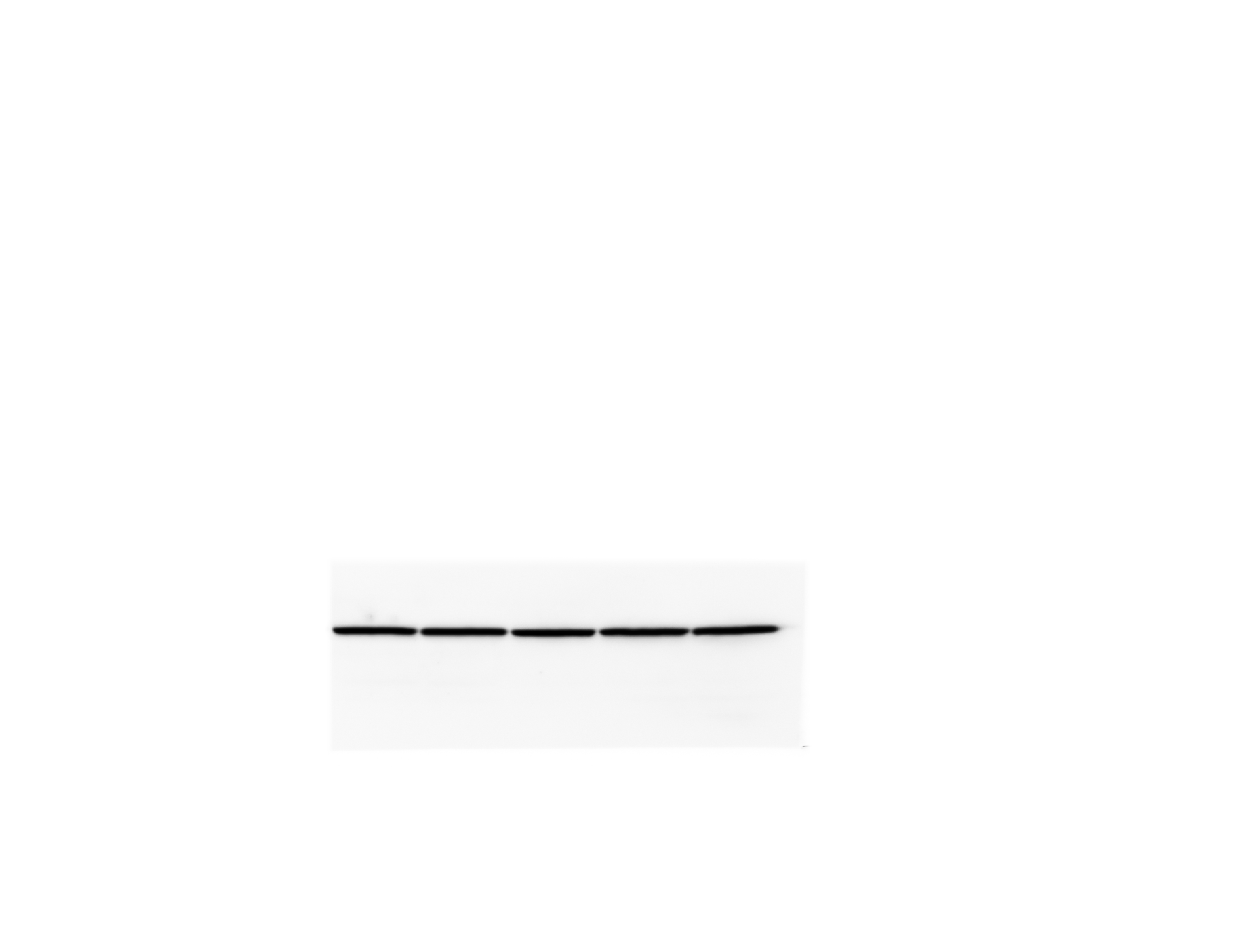

Supplement: Supplementary file 2 [file Data_Sheet_1.ZIP › Original WB images/FIG2/E/GAPDH.tif]

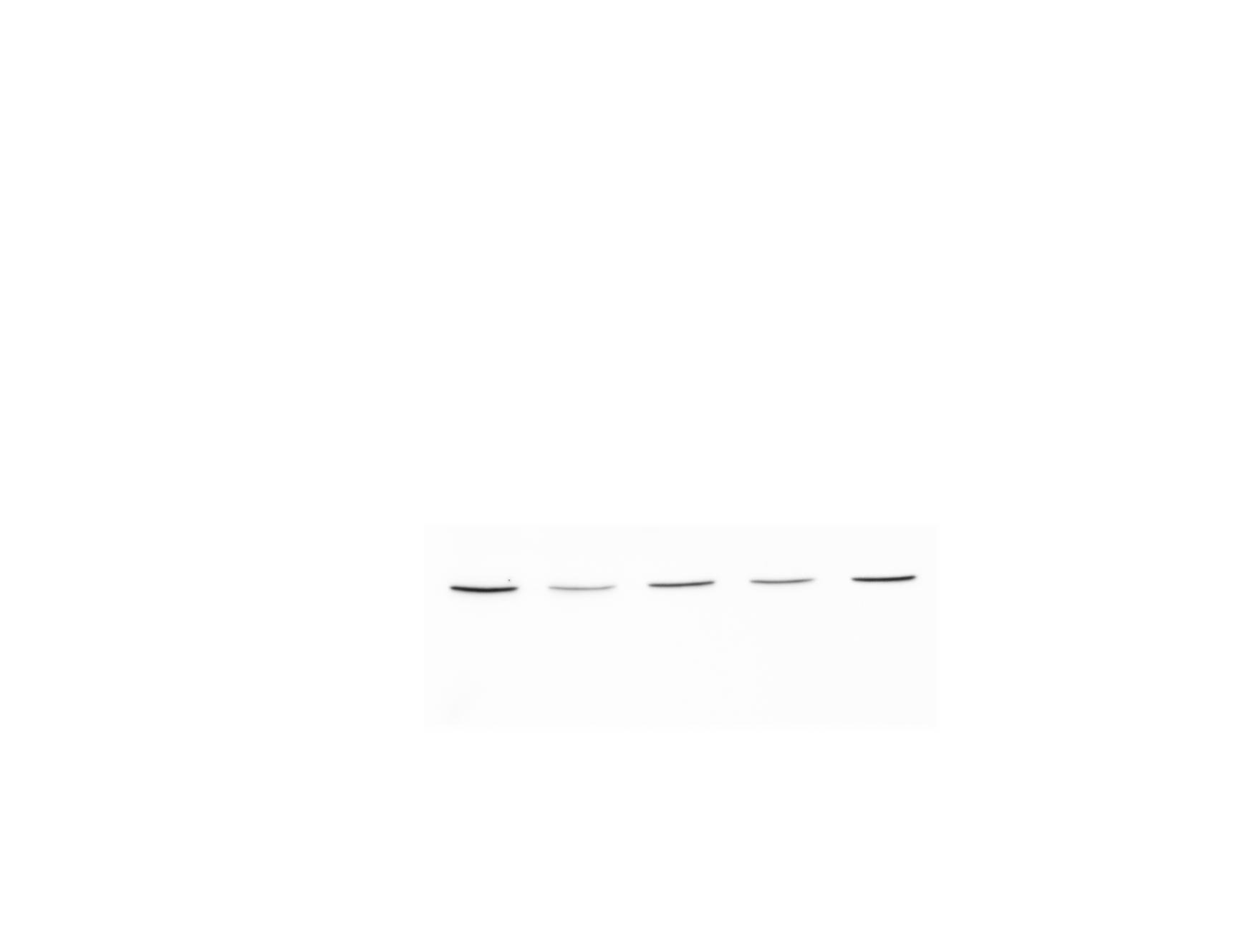

Supplement: Supplementary file 2 [file Data_Sheet_1.ZIP › Original WB images/FIG2/E/TIMP-1.tif]

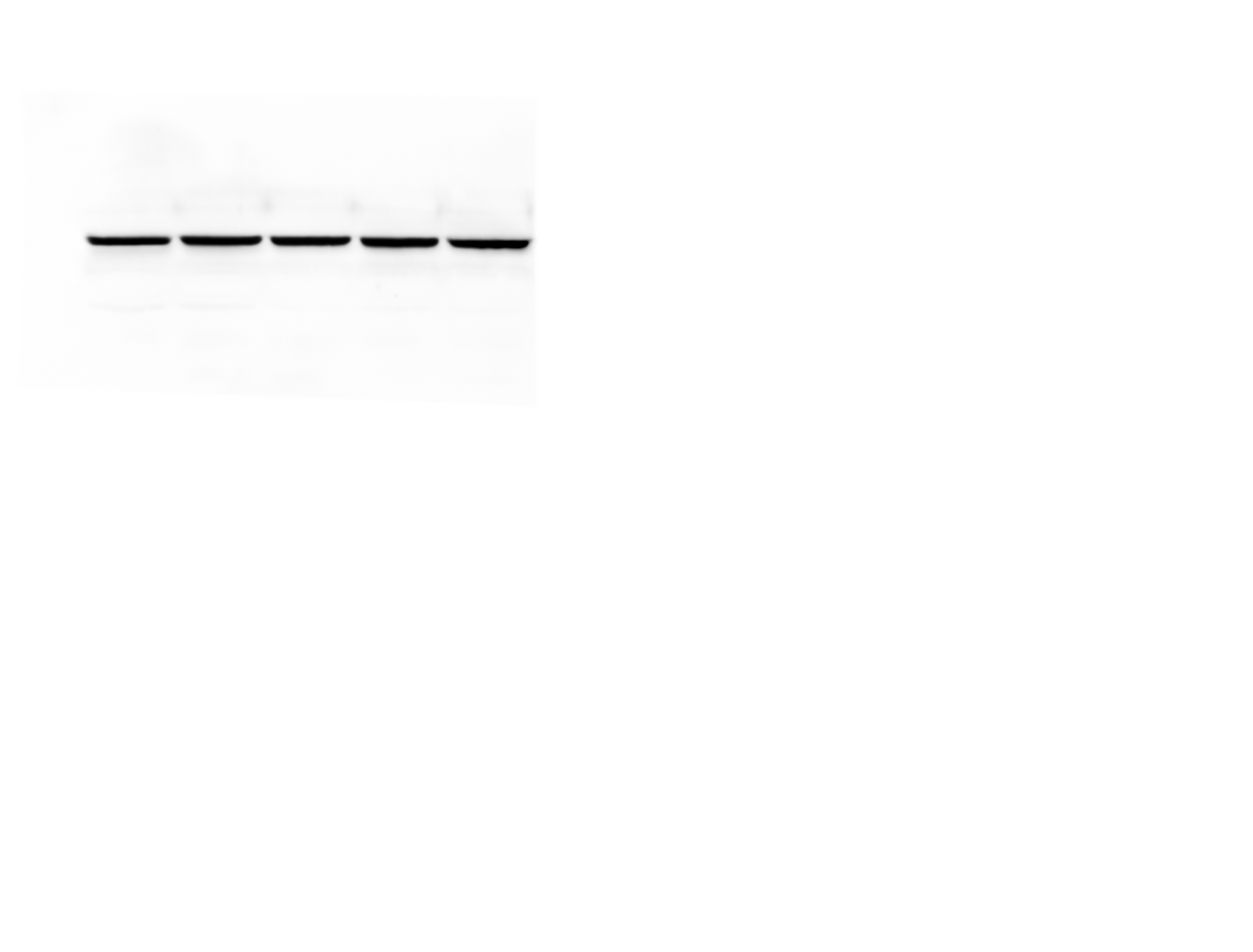

Supplement: Supplementary file 2 [file Data_Sheet_1.ZIP › Original WB images/FIG2/F/GAPDH.tif]

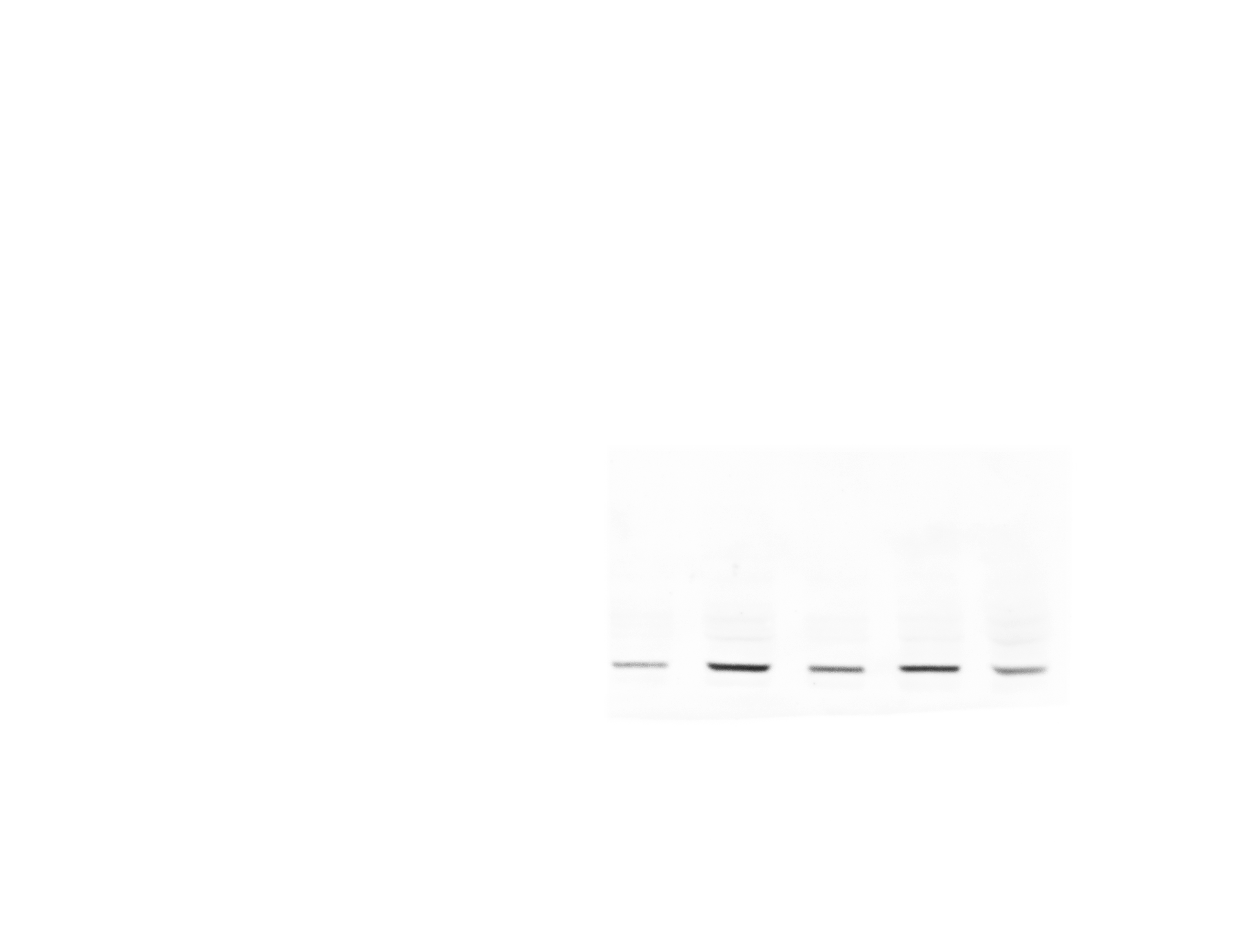

Supplement: Supplementary file 2 [file Data_Sheet_1.ZIP › Original WB images/FIG2/F/MMP-3.tif]

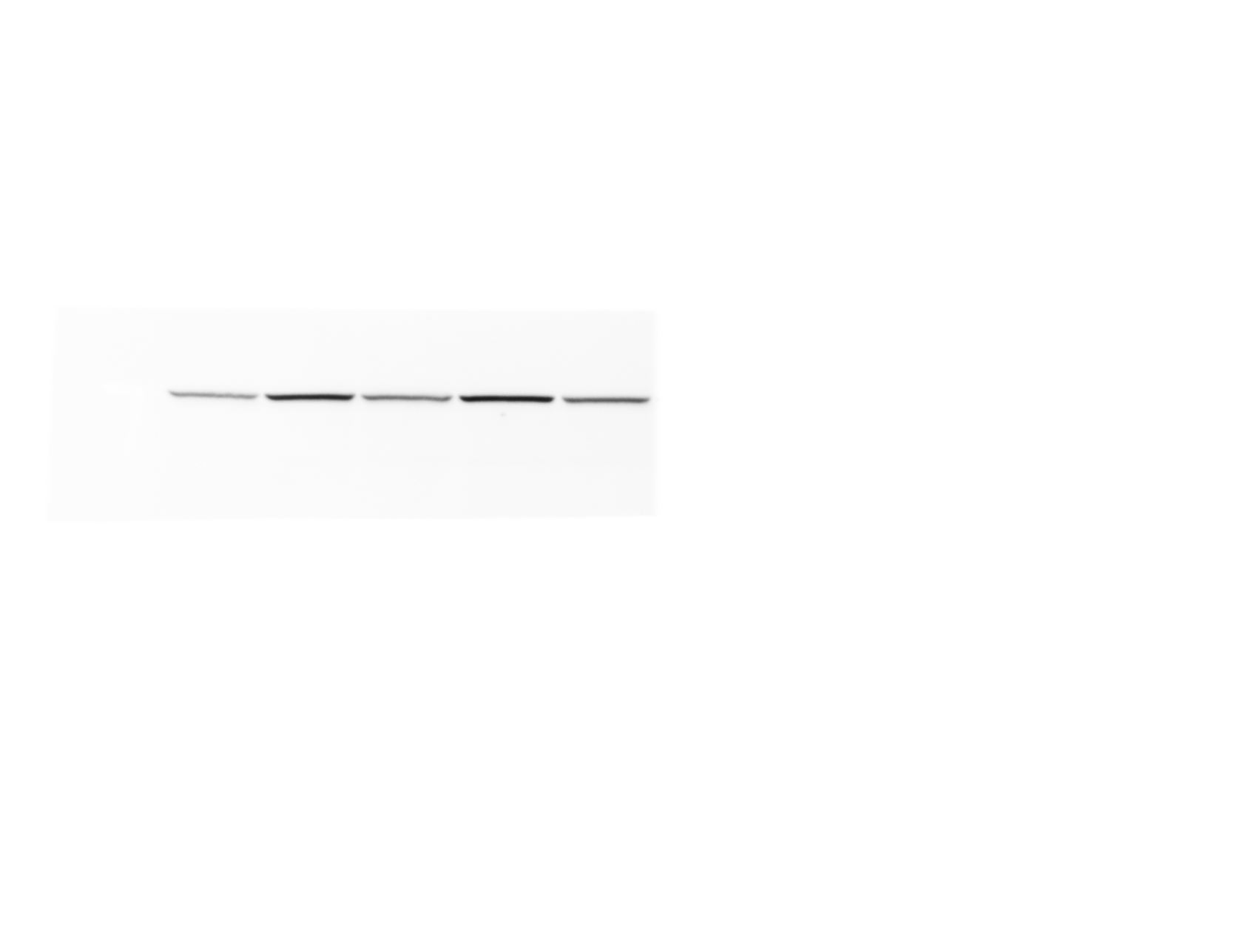

Supplement: Supplementary file 2 [file Data_Sheet_1.ZIP › Original WB images/FIG3/A/BAX.tif]

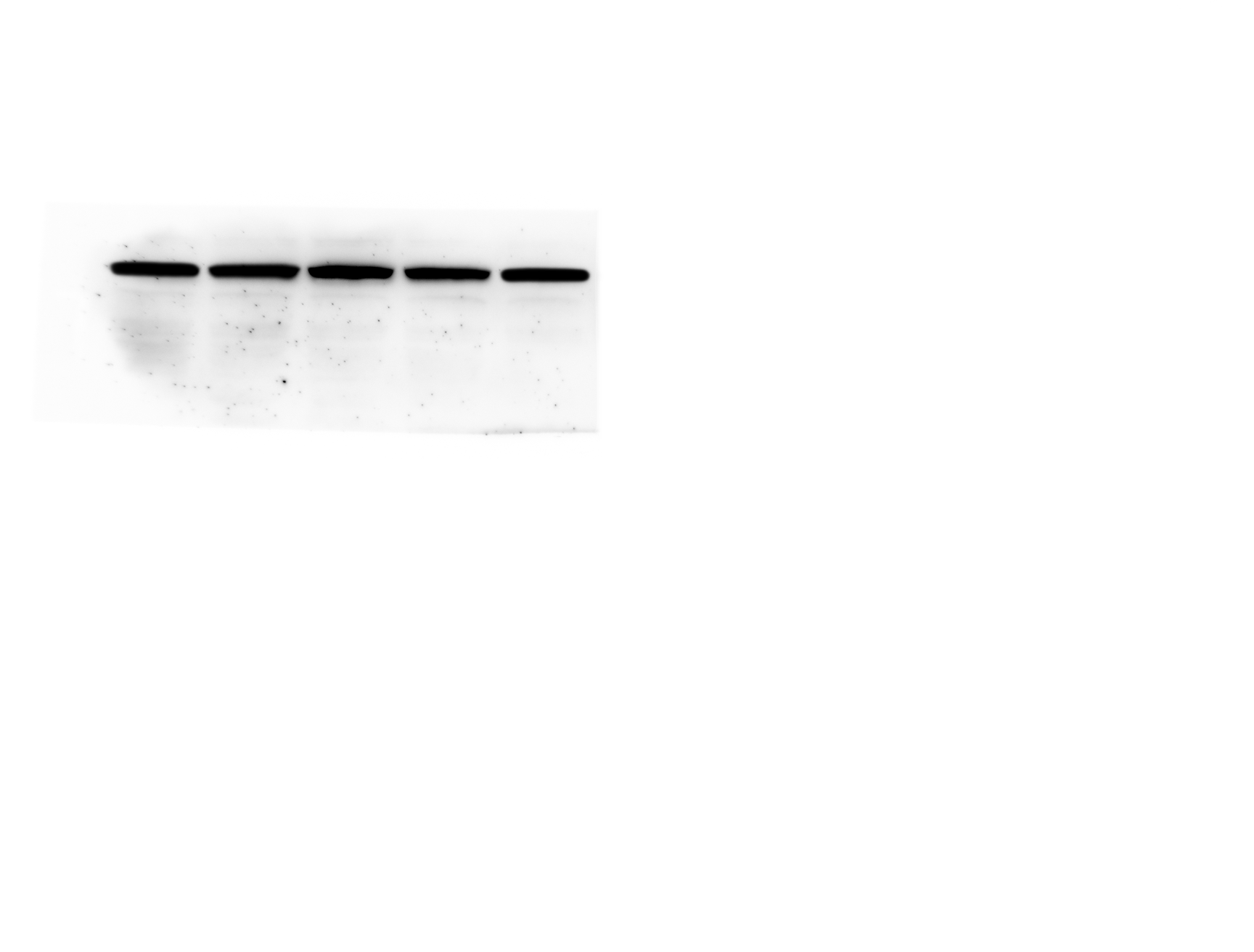

Supplement: Supplementary file 2 [file Data_Sheet_1.ZIP › Original WB images/FIG3/A/GAPDH.tif]

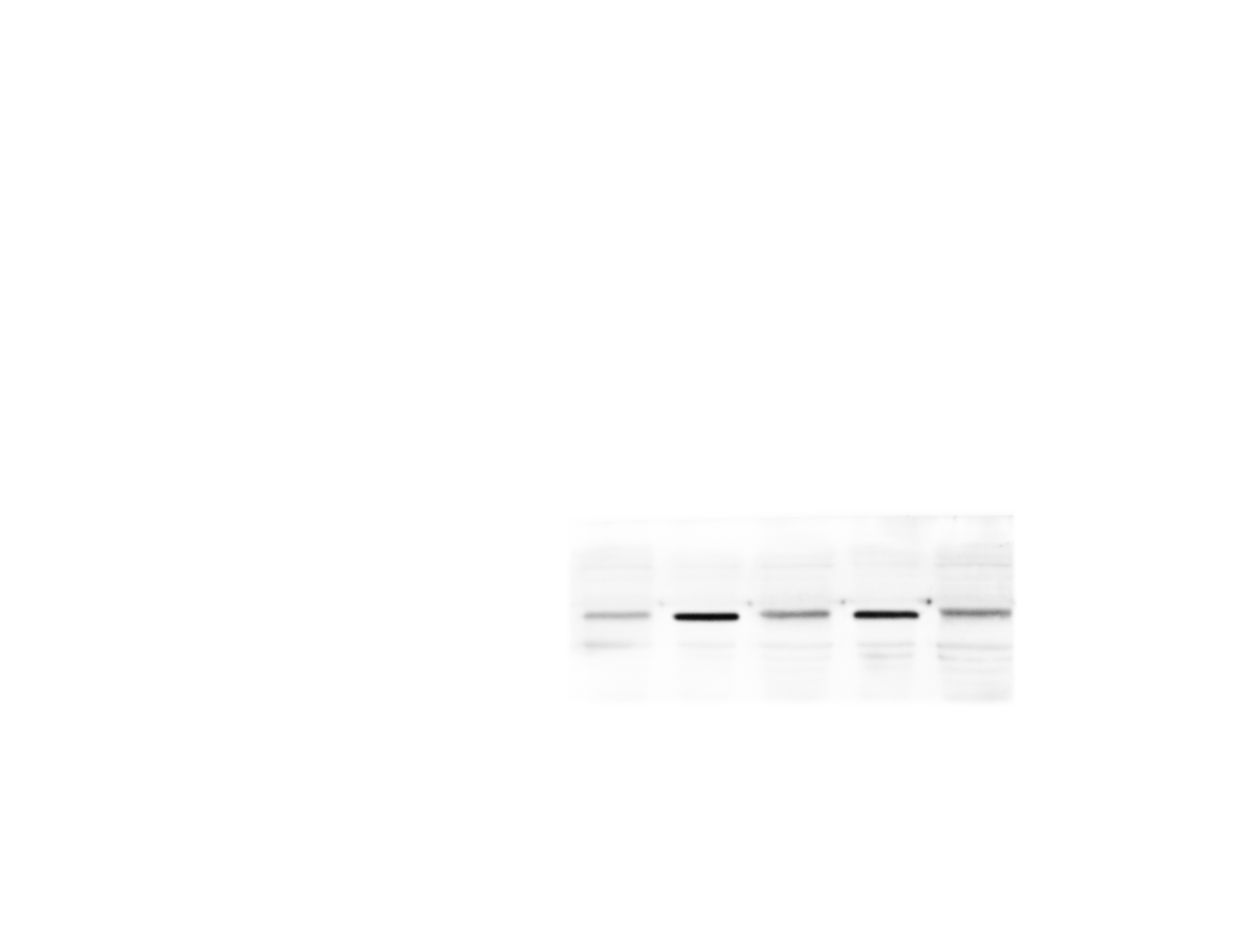

Supplement: Supplementary file 2 [file Data_Sheet_1.ZIP › Original WB images/FIG3/B/BAX.tif]

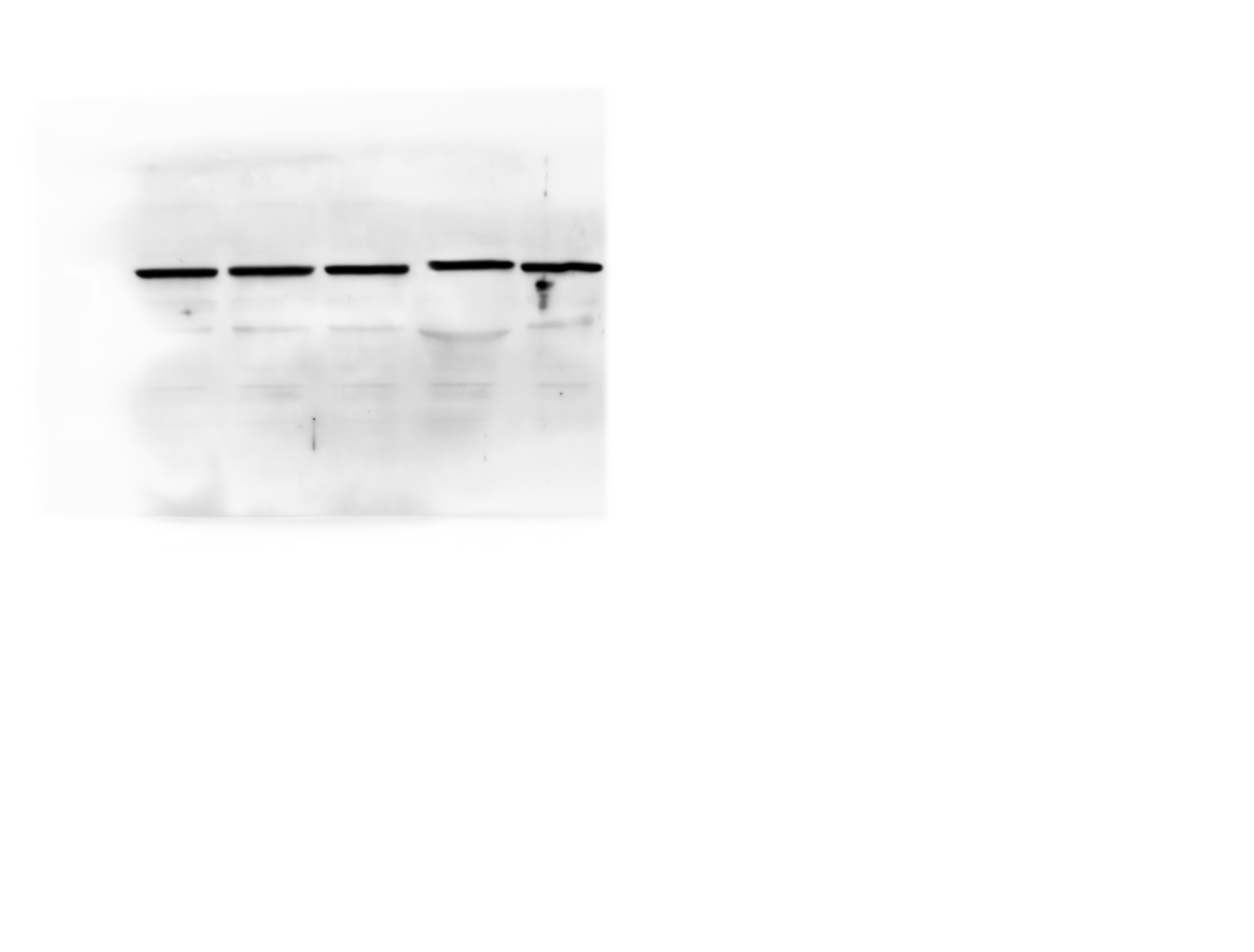

Supplement: Supplementary file 2 [file Data_Sheet_1.ZIP › Original WB images/FIG3/B/GAPDH.tif]

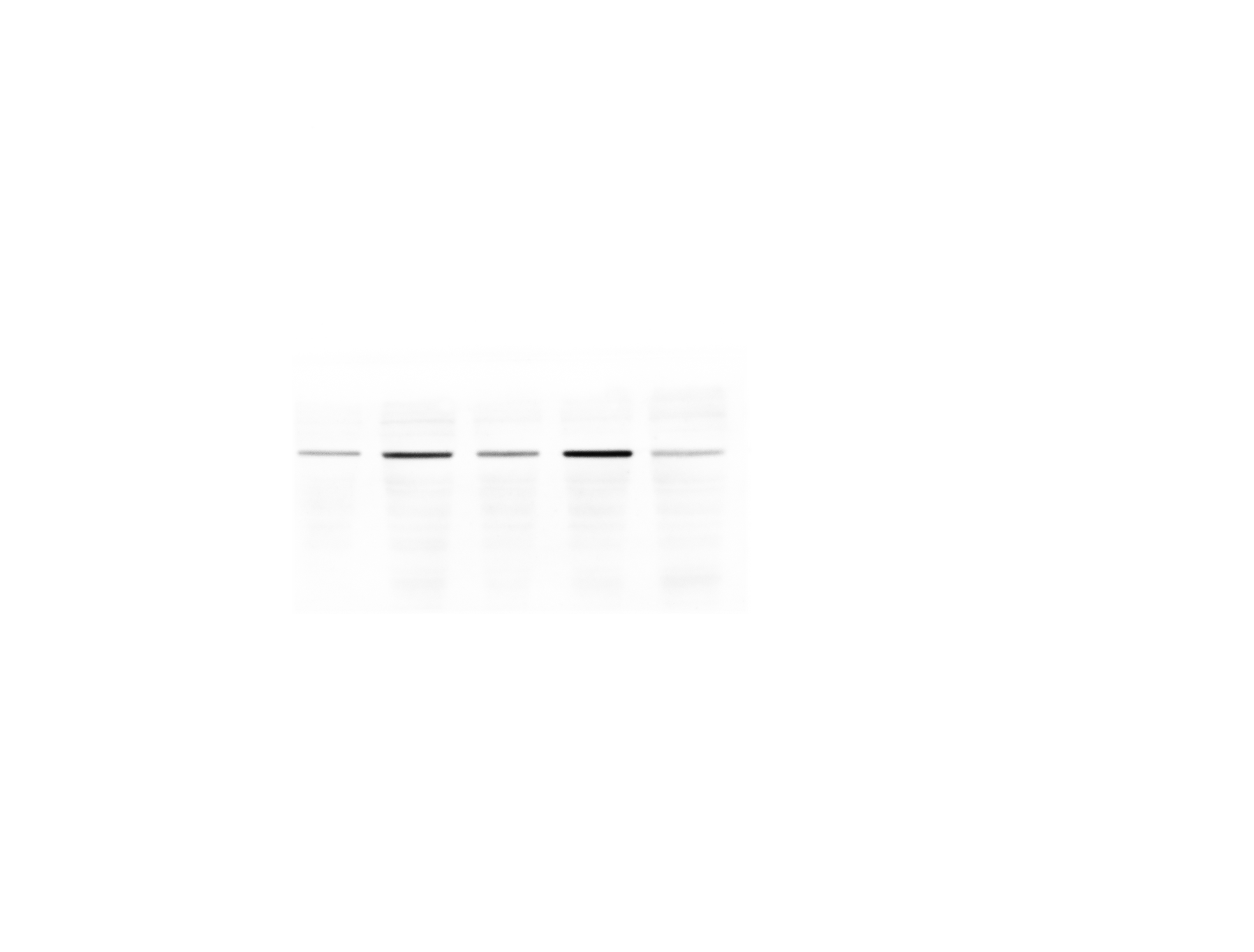

Supplement: Supplementary file 2 [file Data_Sheet_1.ZIP › Original WB images/FIG3/C/BAX.tif]

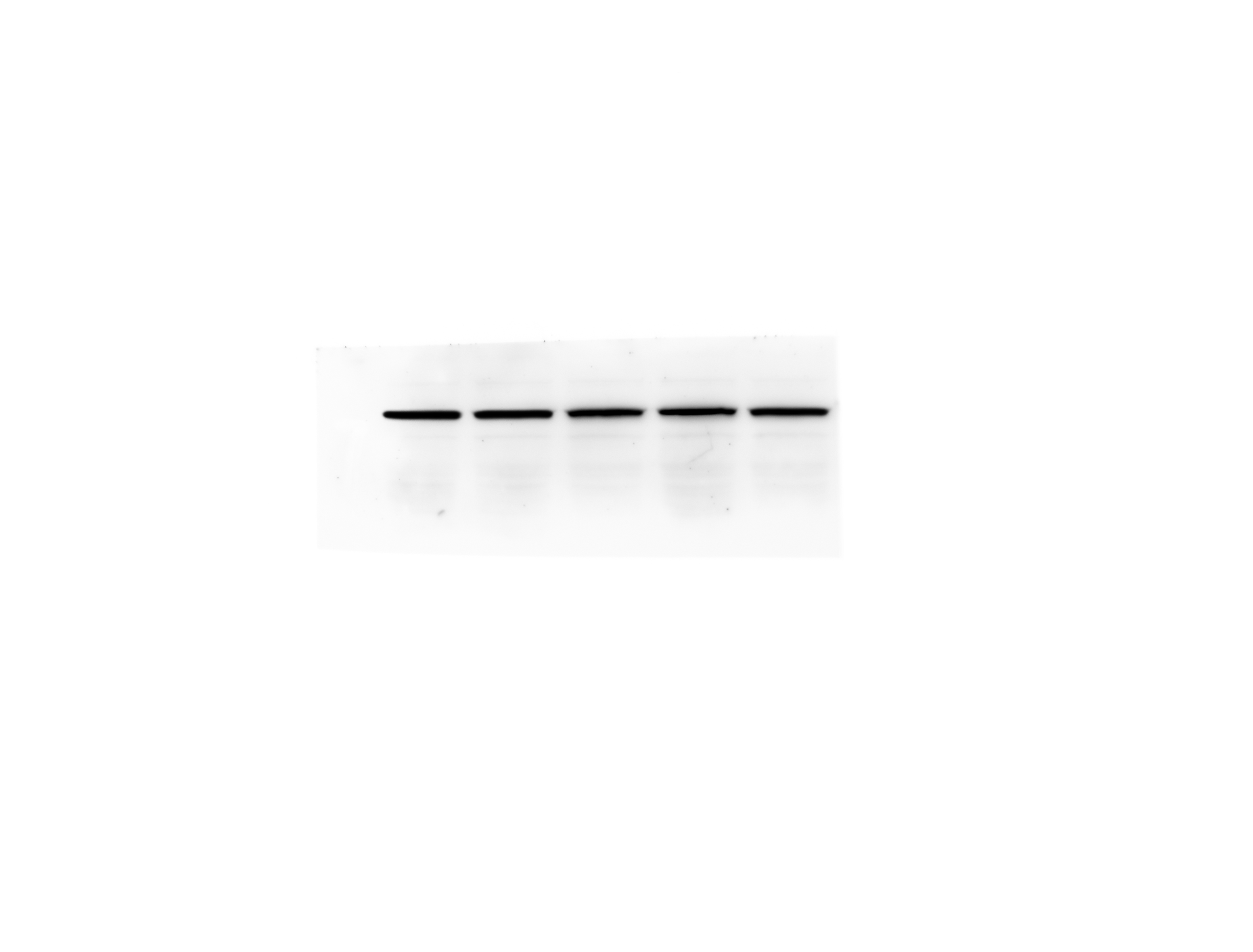

Supplement: Supplementary file 2 [file Data_Sheet_1.ZIP › Original WB images/FIG3/C/GAPDH.tif]

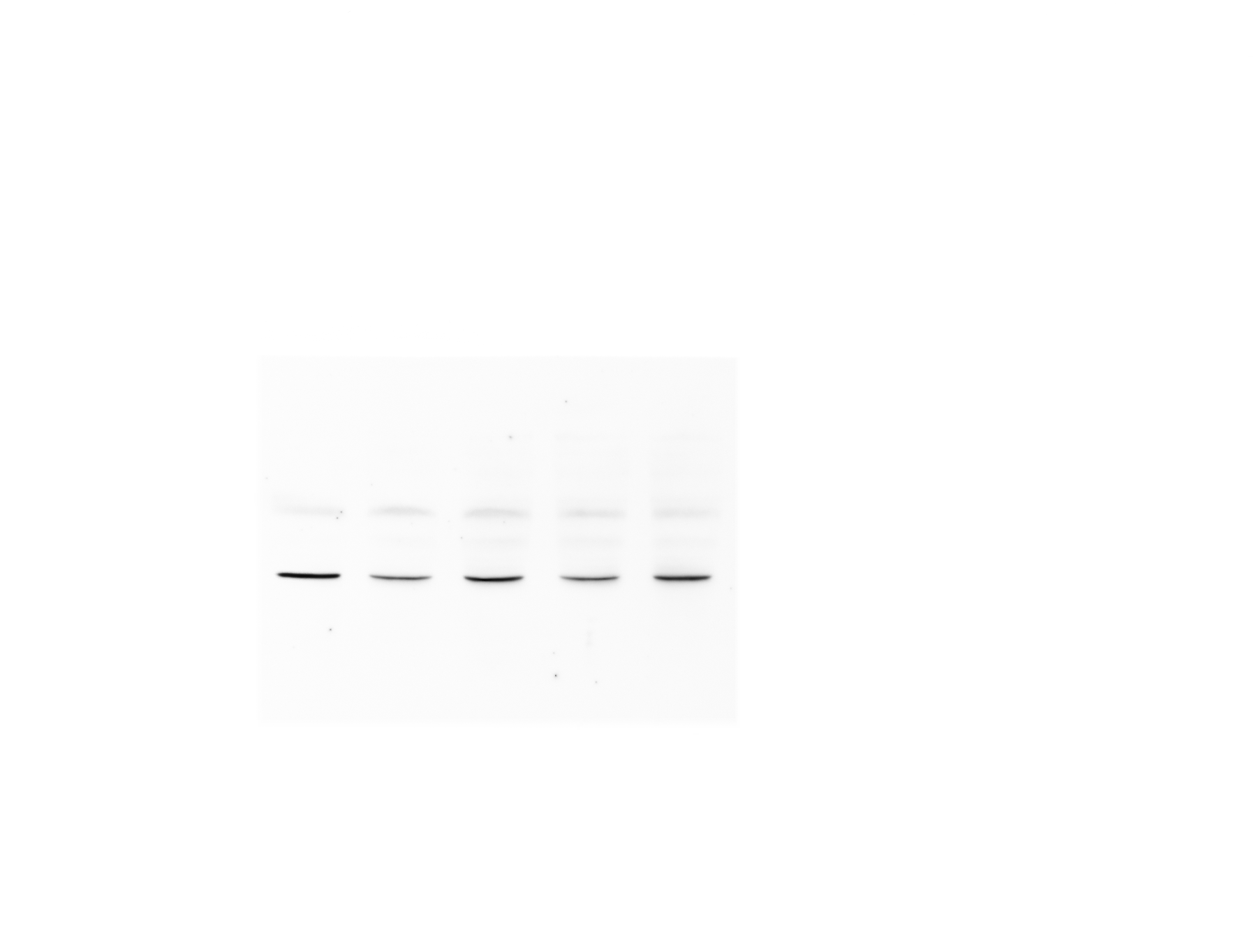

Supplement: Supplementary file 2 [file Data_Sheet_1.ZIP › Original WB images/FIG3/E/Collagen II.tif]

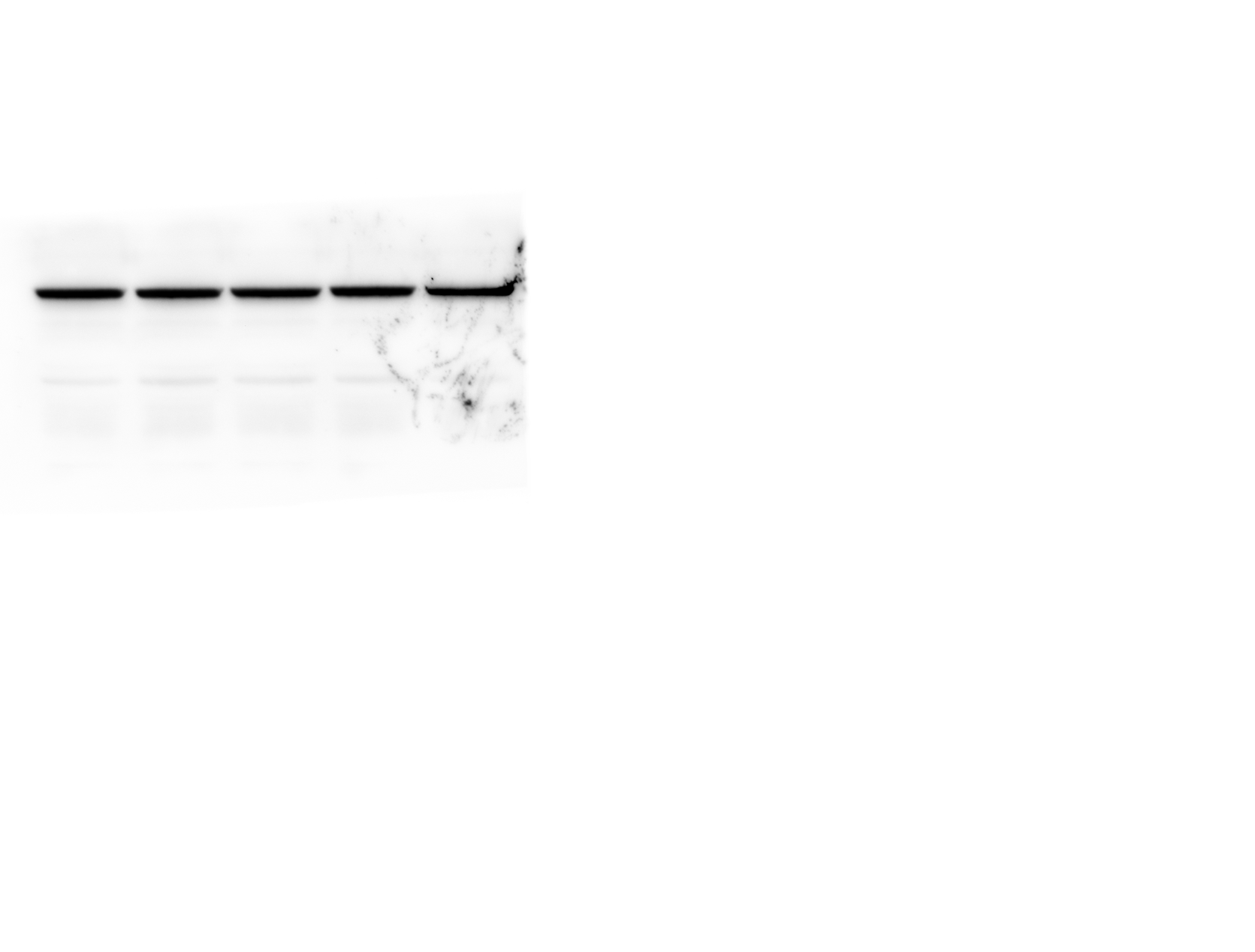

Supplement: Supplementary file 2 [file Data_Sheet_1.ZIP › Original WB images/FIG3/E/GAPDH.tif]

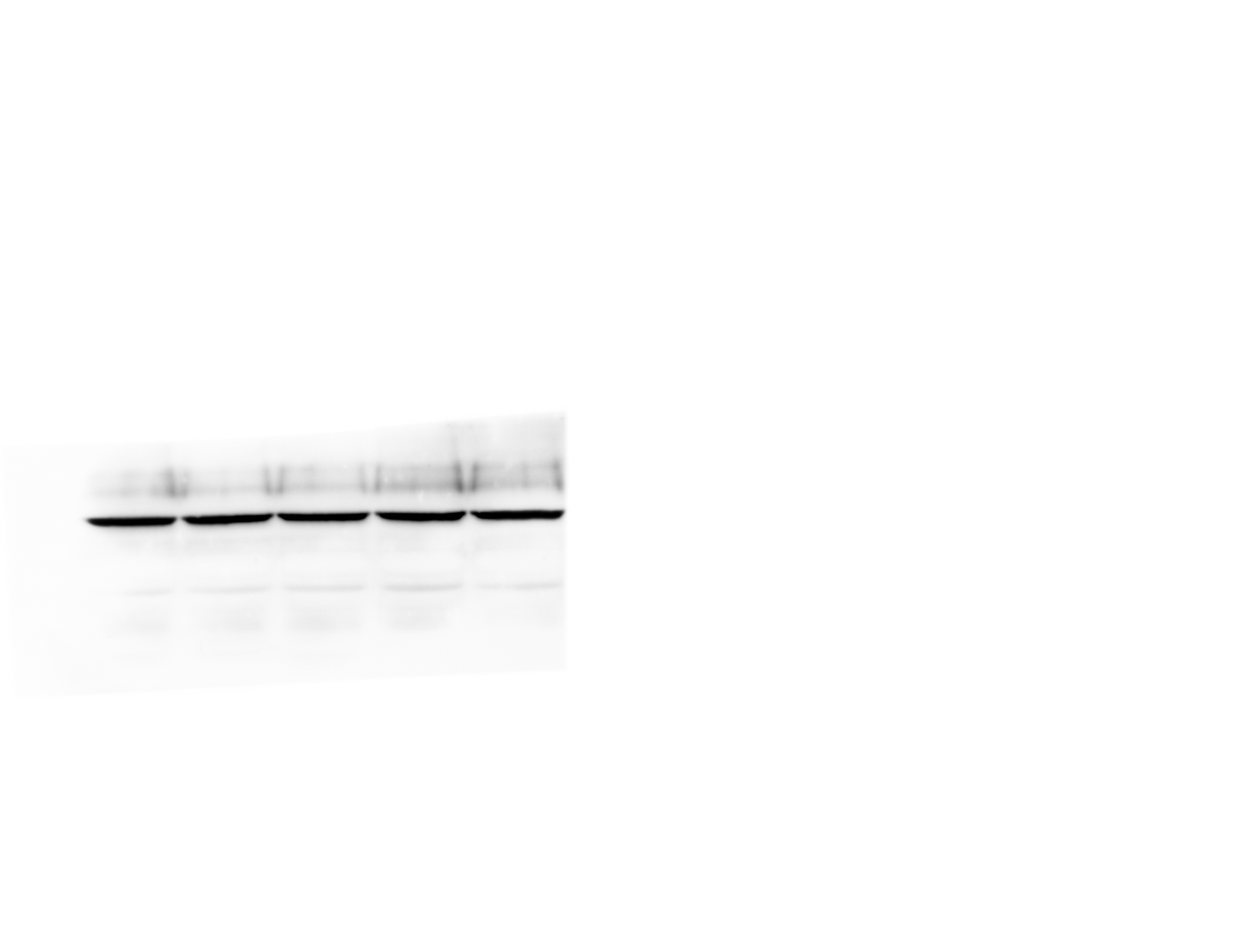

Supplement: Supplementary file 2 [file Data_Sheet_1.ZIP › Original WB images/FIG3/F/GAPDH.tif]

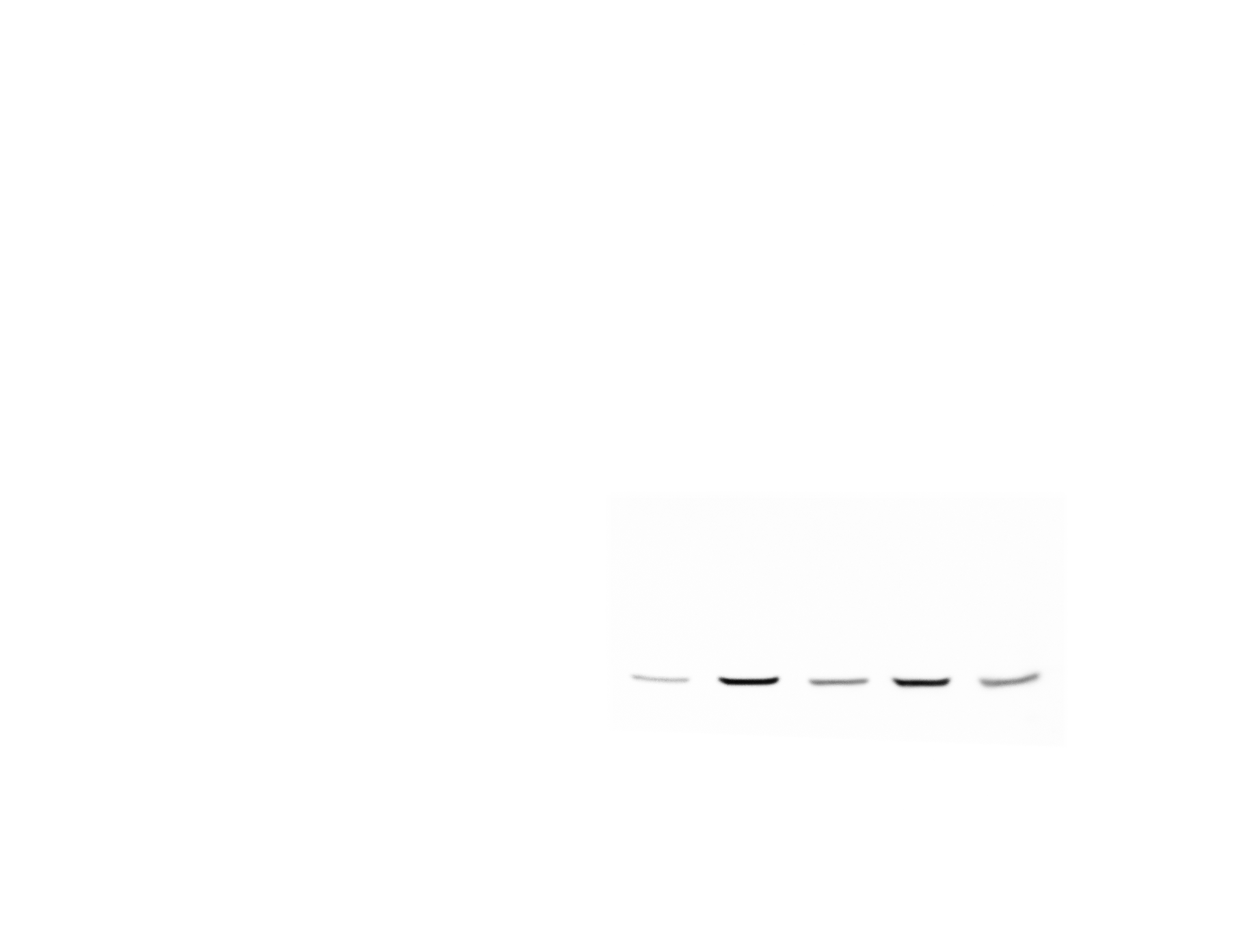

Supplement: Supplementary file 2 [file Data_Sheet_1.ZIP › Original WB images/FIG3/F/MMP-3.tif]

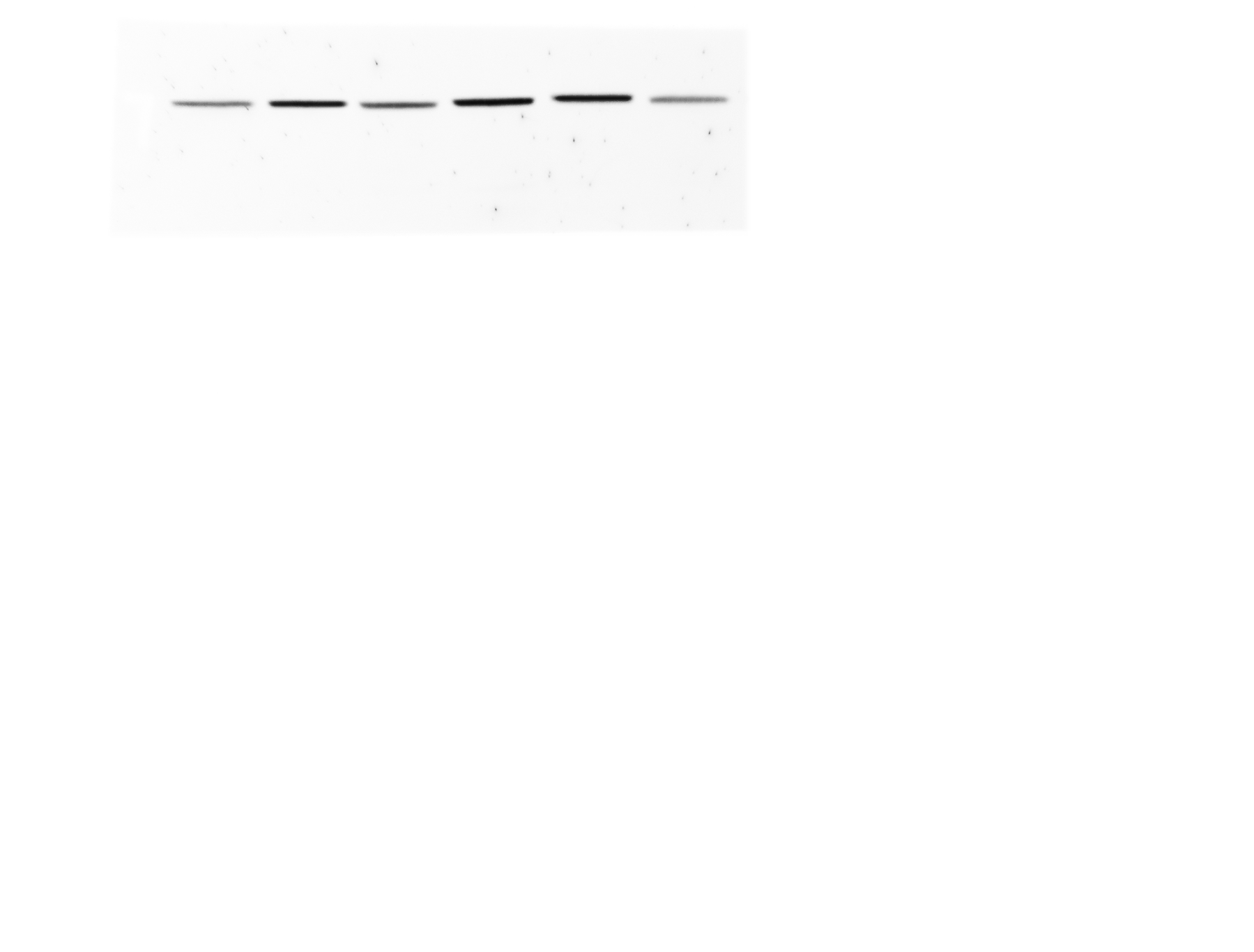

Supplement: Supplementary file 2 [file Data_Sheet_1.ZIP › Original WB images/FIG4/C/BAX.tif]

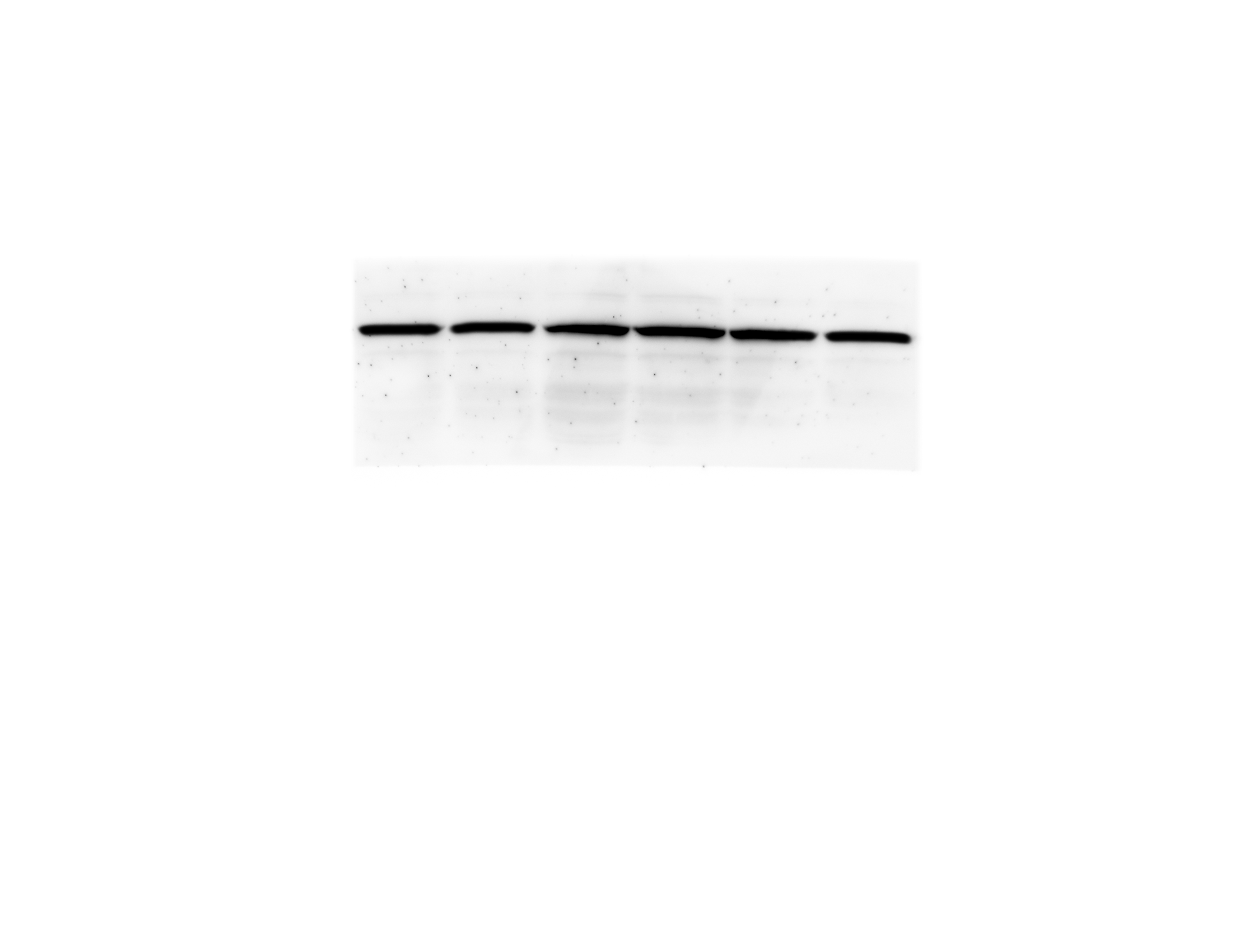

Supplement: Supplementary file 2 [file Data_Sheet_1.ZIP › Original WB images/FIG4/C/GAPDH.tif]

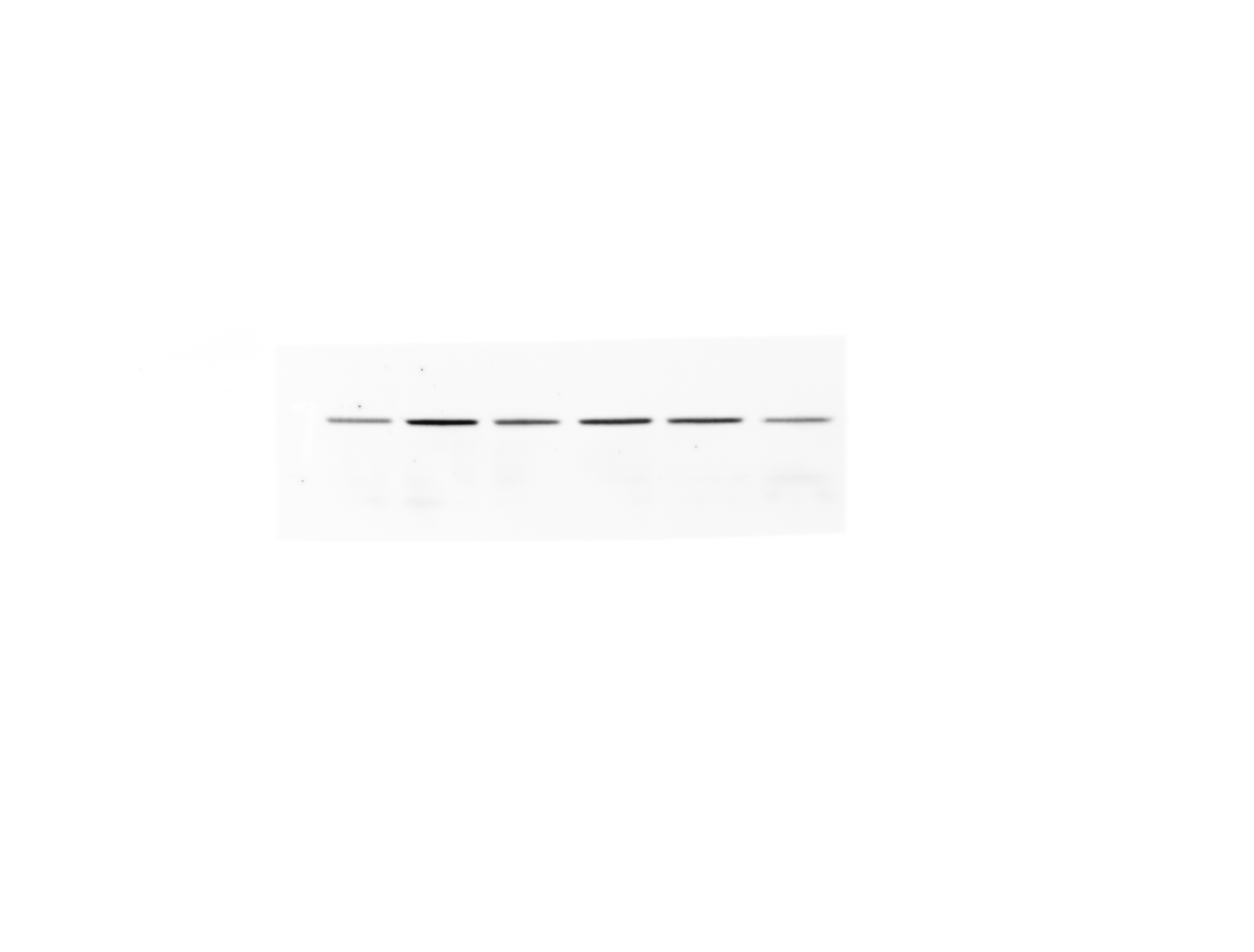

Supplement: Supplementary file 2 [file Data_Sheet_1.ZIP › Original WB images/FIG4/D/BAX.tif]

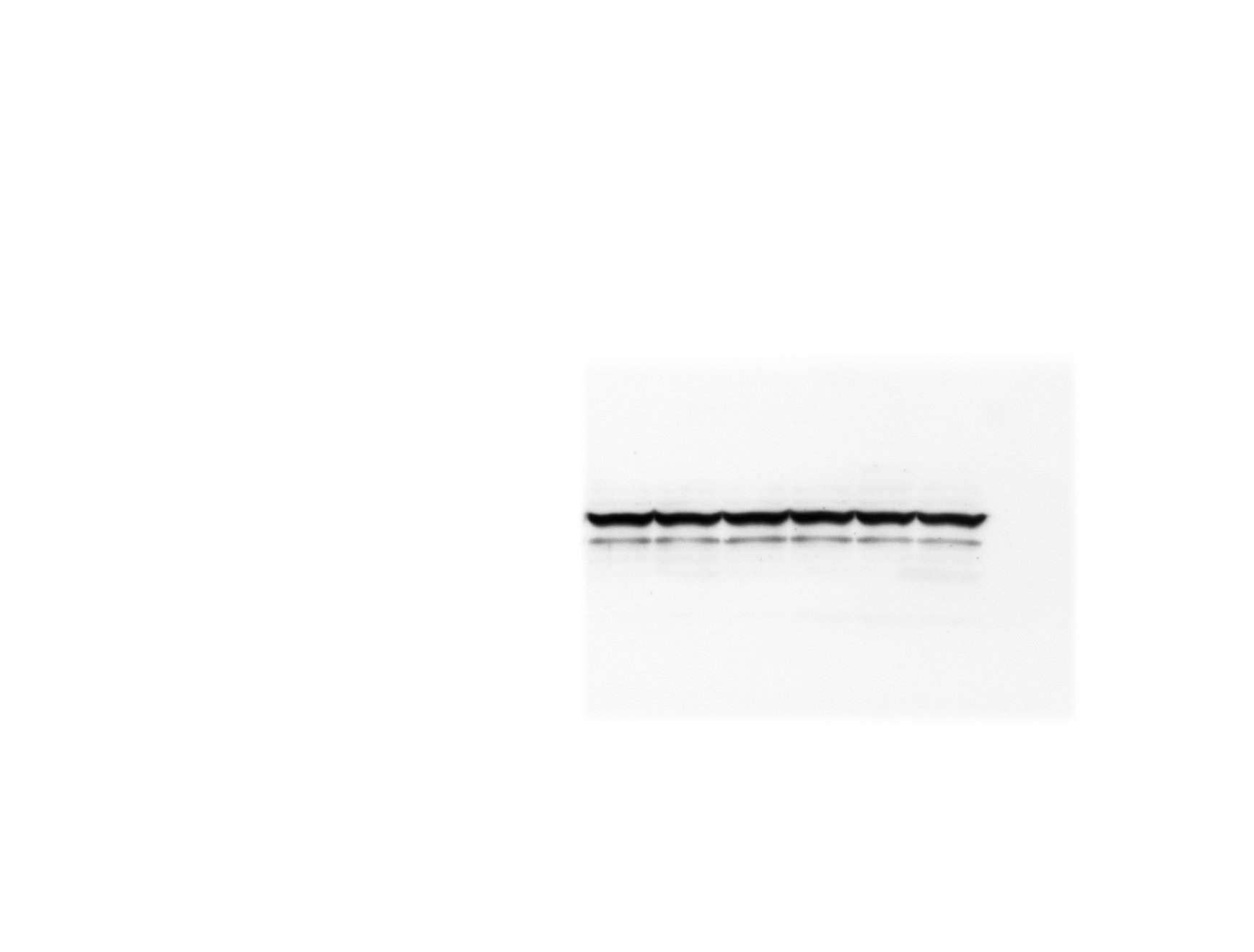

Supplement: Supplementary file 2 [file Data_Sheet_1.ZIP › Original WB images/FIG4/D/GAPDH.tif]

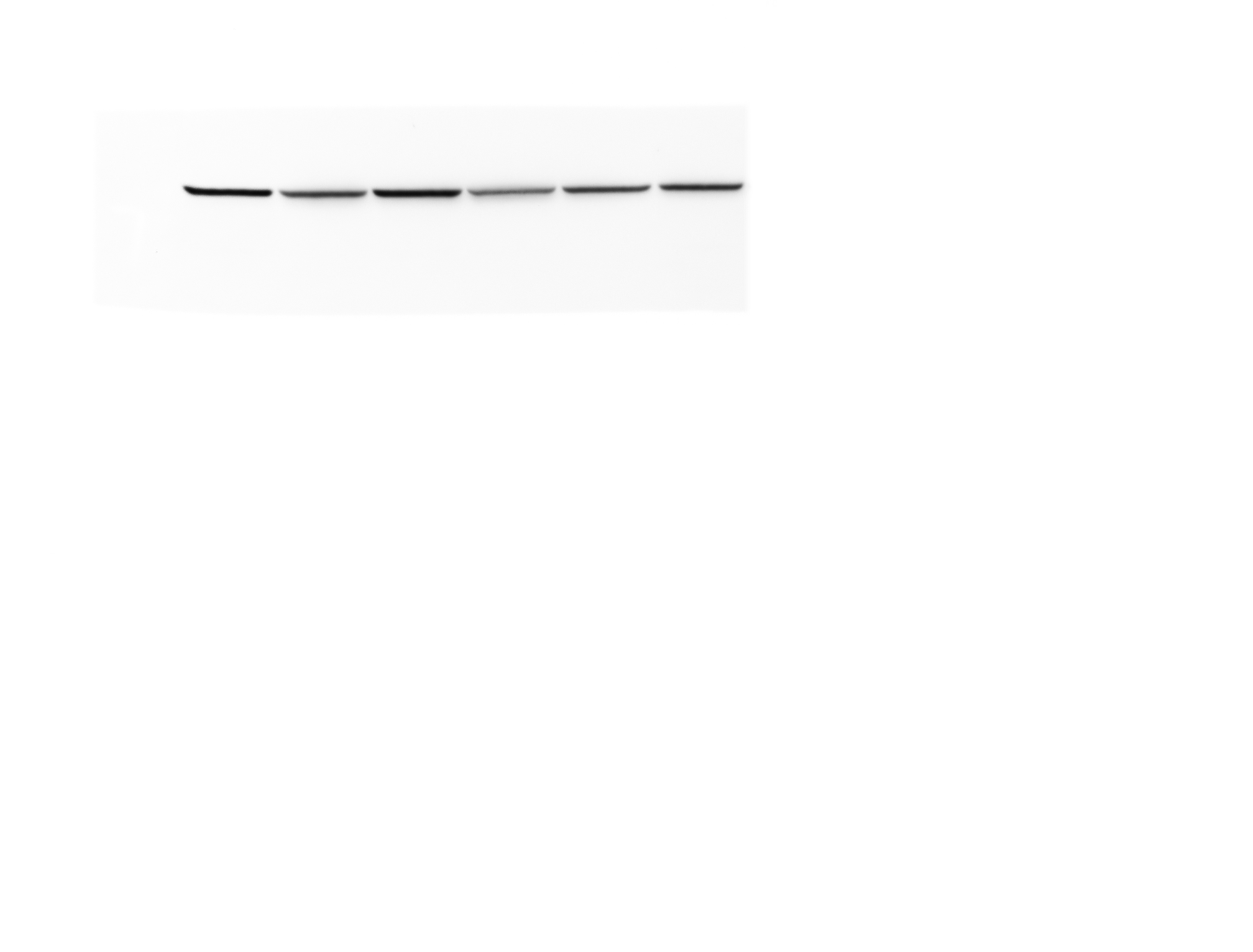

Supplement: Supplementary file 2 [file Data_Sheet_1.ZIP › Original WB images/FIG5/A/Collagen II.tif]

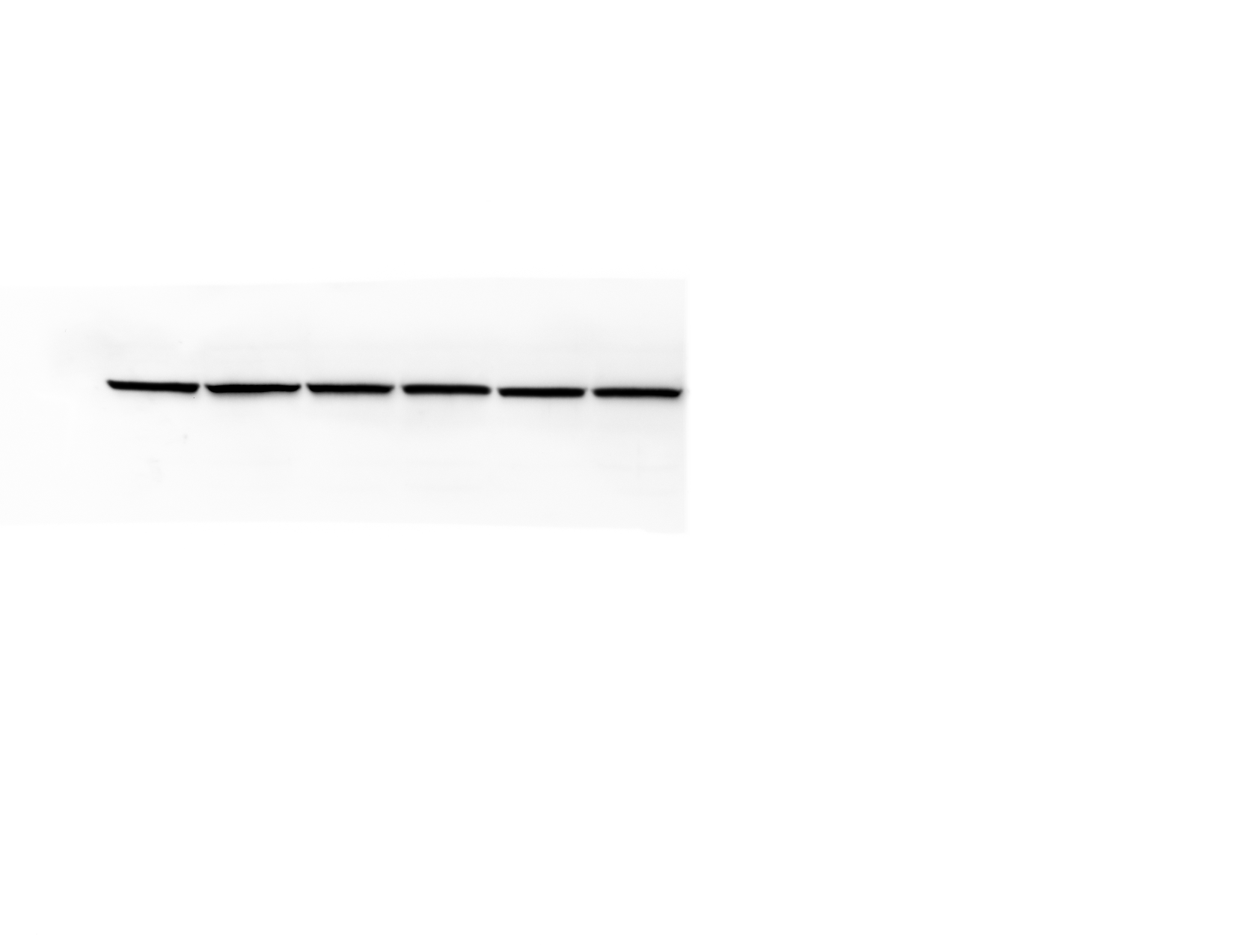

Supplement: Supplementary file 2 [file Data_Sheet_1.ZIP › Original WB images/FIG5/A/GAPDH 1.tif]

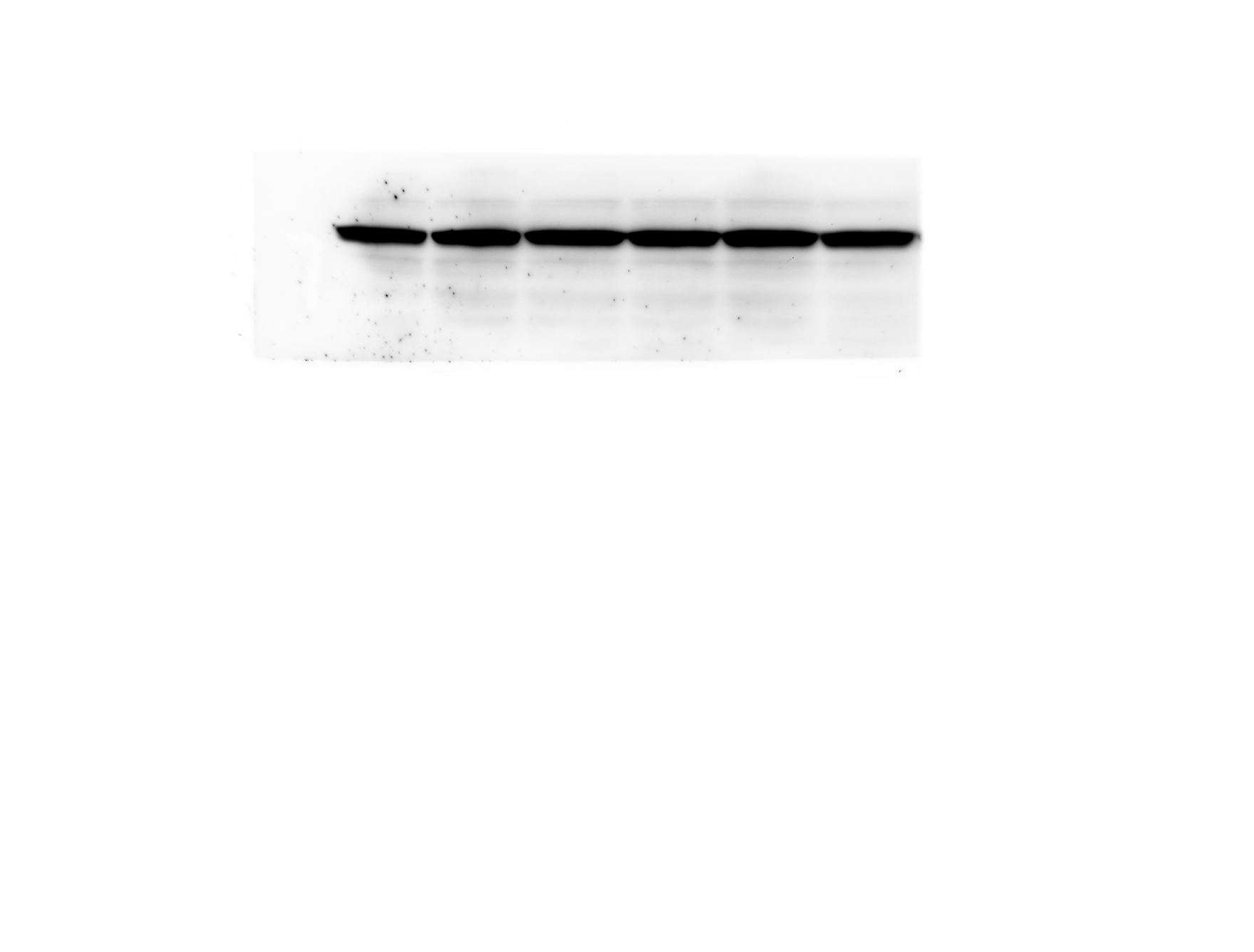

Supplement: Supplementary file 2 [file Data_Sheet_1.ZIP › Original WB images/FIG5/A/GAPDH 2.tif]

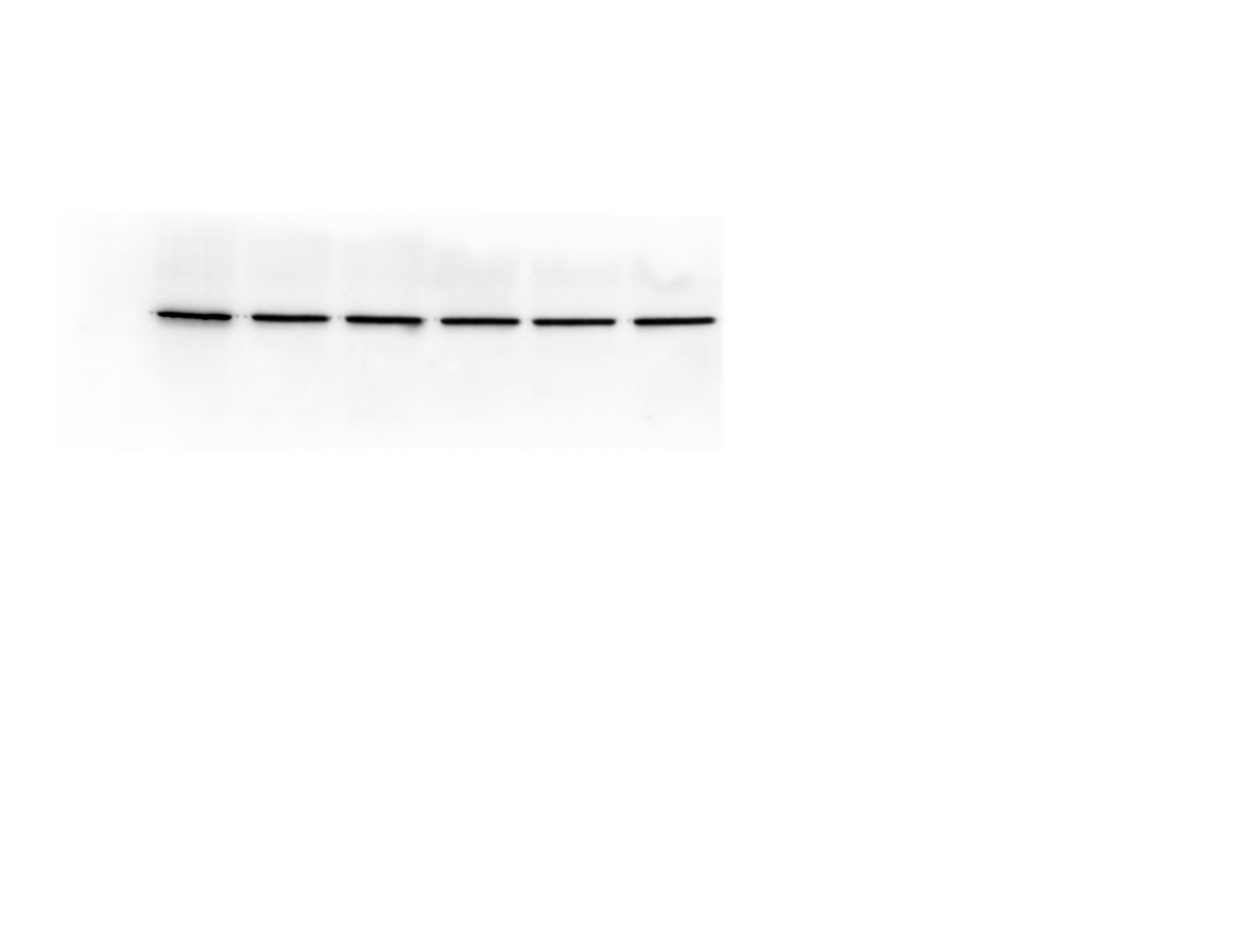

Supplement: Supplementary file 2 [file Data_Sheet_1.ZIP › Original WB images/FIG5/A/GAPDH 3.tif]

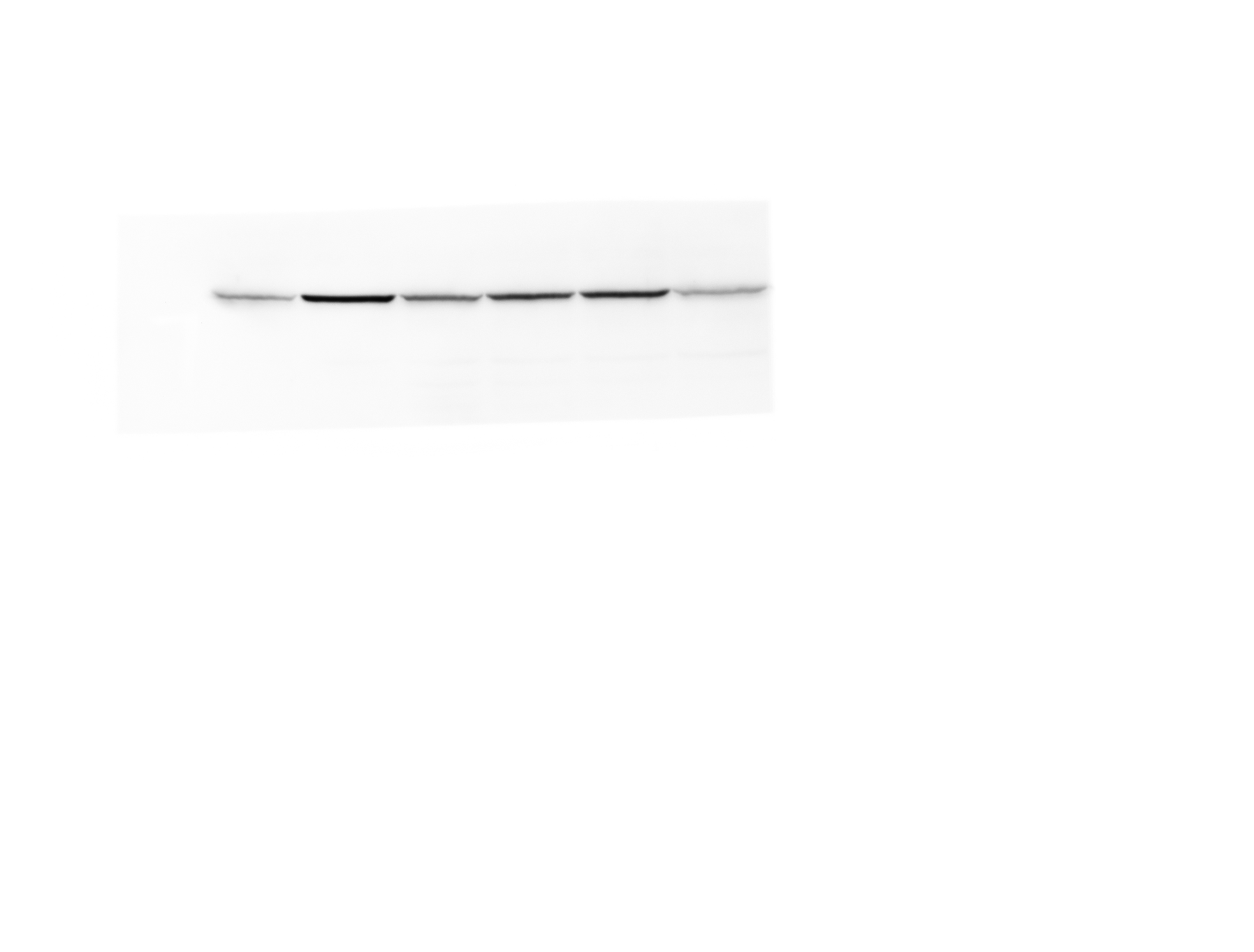

Supplement: Supplementary file 2 [file Data_Sheet_1.ZIP › Original WB images/FIG5/A/MMP-3.tif]

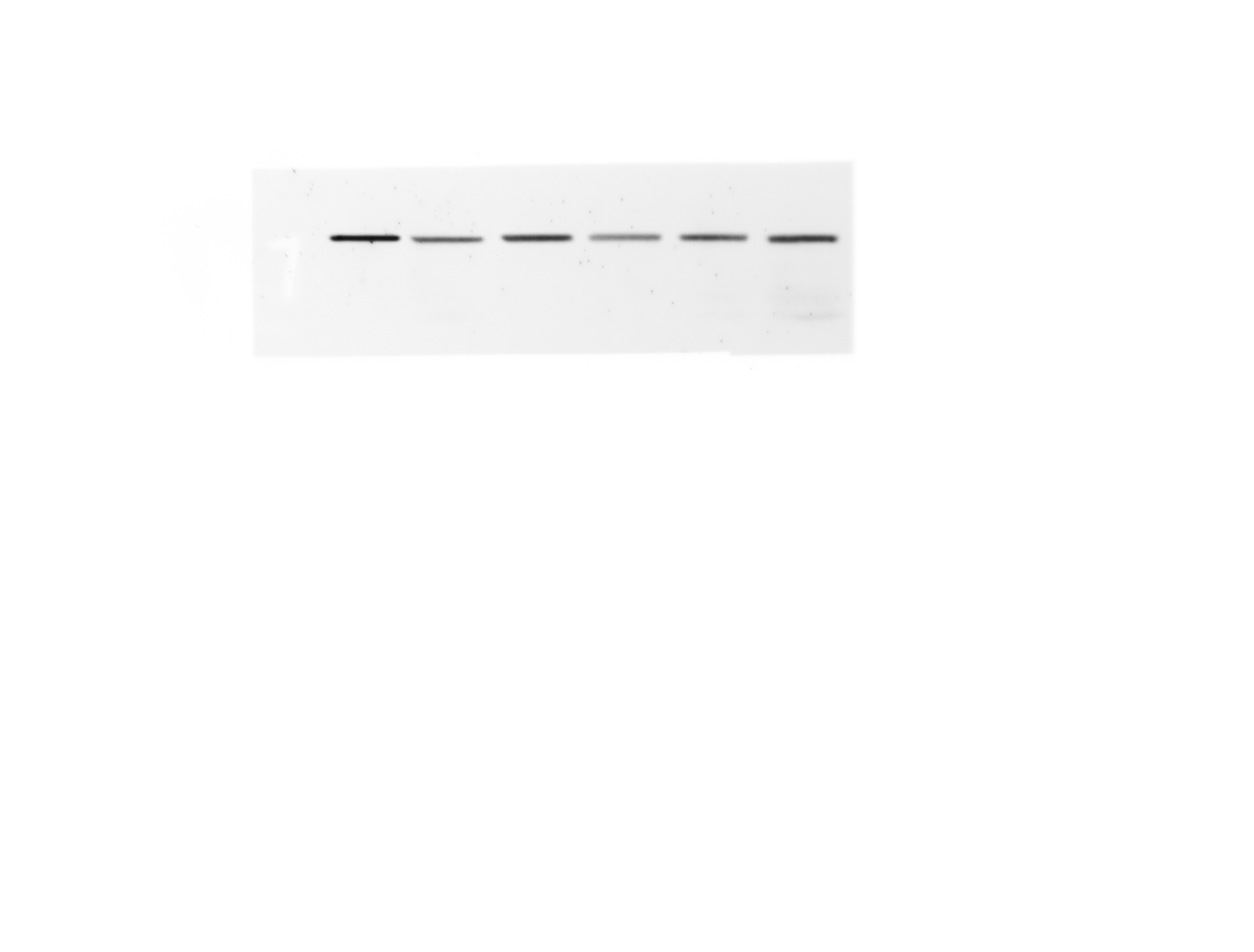

Supplement: Supplementary file 2 [file Data_Sheet_1.ZIP › Original WB images/FIG5/A/TIMP-1.tif]
